# Synthesis and Biological Evaluation of Octahydroquinazolinones as Phospholipase A2, and Protease Inhibitors: Experimental and Theoretical Exploration

**DOI:** 10.3390/molecules28041944

**Published:** 2023-02-17

**Authors:** Md. Afroz Bakht, Thangaiyan Pooventhiran, Renjith Thomas, Mehnaz Kamal, Israf Ud Din, Najeeb Ur Rehman, Imtiaz Ali, Noushin Ajmal, Mohamed Jawed Ahsan

**Affiliations:** 1 Department of Chemistry, College of Science and Humanity Studies, Prince Sattam Bin Abdulaziz University, Al-Kharj 11942, Saudi Arabia; 2 Department of Chemistry, St Berchmans College (Autonomous), Mahatma Gandhi University, Changanassery 686101, Kerala, India; 3 Department of Mechanical Engineering, University Centre for Research & Development, Chandigarh University, Gharuan, Mohali 140413, Punjab, India; 4 Department of Pharmaceutical Chemistry, College of Pharmacy, Prince Sattam Bin Abdulaziz University, Al-Kharj 11942, Saudi Arabia; 5 Department of Pharmacology & Toxicology, College of Pharmacy, Prince Sattam Bin Abdulaziz University, Al-Kharj 11942, Saudi Arabia; 6 Preparatory College, Prince Sattam Bin Abdulaziz University, Al-Kharj 11942, Saudi Arabia; 7 Department of Basic Science and Humanities, Pratap University, Jaipur 303104, Rajasthan, India; 8 Department of Pharmaceutical Chemistry, Maharishi Arvind College of Pharmacy, Ambabari Circle, Jaipur 302039, Rajasthan, India

**Keywords:** octahydroquinazolinone, phospholipase A2, protease activity, DFT study, molecular docking

## Abstract

Phospholipase A2 (PLA2) promotes inflammation via lipid mediators and releases arachidonic acid (AA), and these enzymes have been found to be elevated in a variety of diseases, including rheumatoid arthritis, sepsis, and atherosclerosis. The mobilization of AA by PLA2 and subsequent synthesis of prostaglandins are regarded as critical events in inflammation. Inflammatory processes may be treated with drugs that inhibit PLA2, thereby blocking the COX and LOX pathways in the AA cascade. To address this issue, we report herein an efficient method for the synthesis of a series of octahydroquinazolinone compounds (**4a**–**h**) in the presence of the catalyst Pd-HPW/SiO_2_ and their phospholipase A2, as well as protease inhibitory activities. Among eight compounds, two of them exhibited overwhelming results against PLA2 and protease. By using FT-IR, Raman, NMR, and mass spectroscopy, two novel compounds were thoroughly studied. After carefully examining the SAR of the investigated compounds against these enzymes, it was found that compounds (**4a**, **4b**) containing both electron-donating and electron-withdrawing groups on the phenyl ring exhibited higher activity than compounds with only one of these groups. DFT studies were employed to study the electronic nature and reactivity properties of the molecules by optimizing at the BLYP/cc-pVDZ. Natural bond orbitals helped to study the various electron delocalizations in the molecules, and the frontier molecular orbitals helped with the reactivity and stability parameters. The nature and extent of the expressed biological activity of the molecule were studied using molecular docking with human non-pancreatic secretory phospholipase A2 (hnps-PLA2) (PDB ID: 1DB4) and protease K (PDB ID: 2PWB). The drug-ability of the molecule has been tested using ADMET, and pharmacodynamics data have been extracted. Both the compounds qualify for ADME properties and follow Lipinski’s rule of five.

## 1. Introduction

The chemistry of heterocyclic compounds is an area of great interest for organic as well as medicinal chemists because of their tremendous applications based on their strong coordination abilities and high electron-donating nature [1]. Manipulation and deep study of the biology and chemistry of the heterocyclic moieties paved the way for the discovery of several drugs having clinical significance [2,3,4]. A newly synthesized Schiff’s base containing an azo linkage was shown as a carbon steel corrosion inhibitor in 1 M H_2_SO_4_ [2,3,4] and selective naked-eye sensors for acetate anion [5]. Isoenzymes of human carbonic anhydrase (hCA) contain zinc ions, and they are widespread metalloenzymes that play an important role in maintaining pH equilibrium [6].

Heterocyclic structures having a nitrogen bicyclic nucleus such as quinzolines and octahydroquinazolines are the pioneers for the development of a large number of drugs, and several researchers around the world have also focused on other studies [7]. Recently, octahydroquinazoline scaffolds have been reported with a variety of medicinal properties, including anticancer [8], anti-inflammatory [9], and antimicrobial [10]. The acid-catalyzed cyclocondensation of aldehydes, ethylacetoacetate, and urea to produce octahydroquinazoline is a well-known Bigineli type of reaction [11]. HCl, Conc. H_2_SO_4_ [11], and some Lewis acids (La(OTf) [3], L_2_O_3_, ZrCl_4_) [12] were used as catalysts in the synthesis of octahydroquinazoline. These are well-known reagents, but their usage in octahydroquinazoline synthesis is limited due to their low yield, side product generation, and relative cost. The multifunctional polyoxometalates family of acids, notably heteropoly acid (HPA), is more active than other solid acids (SiO_2_, zeolites, and Al_2_O_3_) as a replacement for such acids [13]. Due to its poor thermal stability, HPA has a small surface area and a limited ability to regenerate. Certain transition metals (Pd/Pt) and silicon dioxide were added to HPA to improve the regeneration capacity and increase the surface area, respectively [14]. Phospholipases A2 (PLA2) are secretory enzymes that catalyze the breakdown of membrane glycerophospholipids to liberate fatty acids and lysophospholipids [15]. They are abundantly found in mammals and snake venoms. They play a role in a variety of inflammatory processes, including the production of eicosanoids and lysophospholipids, cellular membrane homeostasis, and lipid digestion [16]. PLA2 is divided into ten categories: IB, IIA, IIC, IID, IIE, IIF, III, V, X, and XIIA [17]. Among these groups, IB PLA2 has been reported to be involved in physiological and pathophysiological processes such as cell migration, proliferation, apoptosis, and hormone release [18]. In addition, PLA2 is also found in body secretions such as pancreatic juices, arthritic synovial fluid [19], and in the serum of patients suffering from lung injury [20] and chronic renal failure [21].

Proteases are proteolytic enzymes that aid in the breakdown of proteins into peptides and amino acids. These enzymes are categorized as Bacillus protease, protease-esperase, and protease K [22] based on their sources and functions. Many pathogenic disorders, such as inflammation, cancer, hypertension, and AIDS, are caused by enzymes derived from plants, animals, and microbes [18]. Protease inhibitors can thus be employed as therapeutic targets in the development of medicines and the prevention of disease. Quinazoline derivatives are being studied to see if they can eliminate the risk factors for colorectal cancer (CRC) and other inflammatory disorders by inhibiting proteases or proteases and phospholipase A2 [23,24,25].

**Figure 1:**
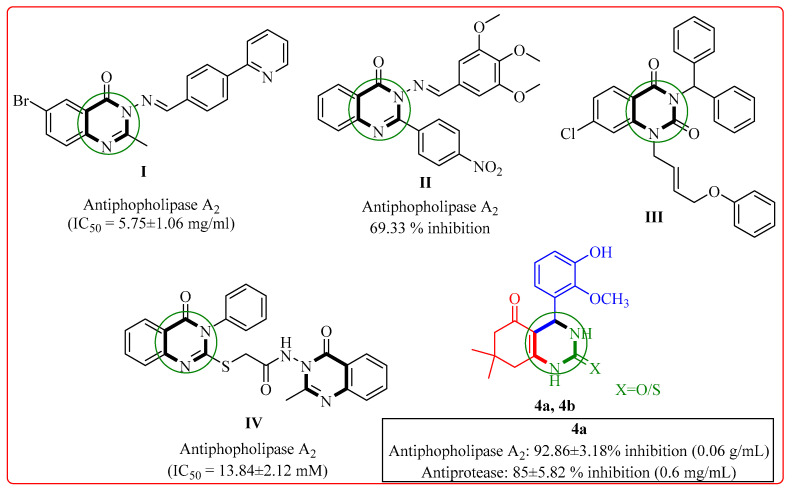
Representative reported compounds (**I**–**IV**) compared with compounds **4a**, **4b** as anti-phospholipase A2, protease activities.

Quinazoline containing compounds such as 3*H*-quinazolin-4-one compounds (**I**) [23], 3-substituted benzylideneamino-2-(4-nitrophenyl) quinazolin-4(3*H*)-ones (**II**) [24], *N*-(aryl)-2-((6methyl/6,7-dimethoxy-4-oxo-3-phenyl (**III**) and benyl-3,4-dihydroquinazolin-2-yl)thio)acetamide (**IV**) derivatives [25] were developed recently as potential antiprotease and antiphospholipase A2 inhibitory activity (Figure 1). We describe here the synthesis of a series of octahydroquinazolinone compounds (**4a**–**h**) and evaluation of their phospholipase A2 and protease activities in continuation of our prior work on the target phospholipase A2 and protease [18]. Furthermore, the antiphospholipase A2 and antiprotease activities of the two compounds were tested. DFT was used to determine structural stability and reactivity, as well as docking and other theoretical studies, which were also performed.

## 2. Results

### 2.1. Chemistry

**Scheme 1:**
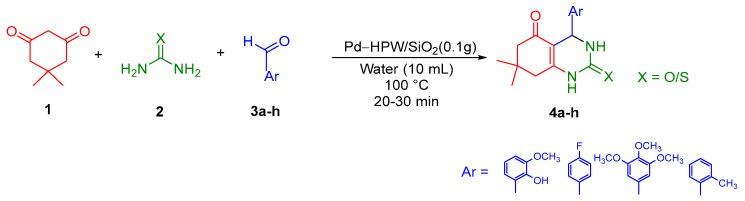
Pd-HPW/SiO_2_ catalyst-mediated octahydroquinazolinone synthesis.

As shown in Scheme 1, compounds were produced utilizing Pd-HPW/SiO_2_ as green catalysts in absolute aqueous conditions. As a result, only two of all synthesized compounds exhibited good biological activities. Therefore, we tested further FT-IR, Raman, mass, and NMR techniques to elucidate newly synthesized compounds spectroscopically.

#### 2.1.1. Spectroscopic Characterization of Synthesized Compounds

##### FT-IR

Compounds **4a** and **4b** were confirmed by FT-IR. The frequencies at 3477 and 3363, and 3390 and 3380 cm^−1^ in FT-IR symmetric stretching N–H vibrations are for compounds **4a** and **4b**, respectively. Peaks at 3191 and 3305 cm^−1^ are attributed to broad OH [26,27]. The C=O ring has a peak between 1683 and 1685 cm^−1^ for both compounds. For **4a** and **4b**, the peaks at 1593 and 1595 belong to amide (CO–NH) and thioamide (C=S–NH), respectively, indicating the synthesis of octahydroquinazolinenone. Another distinctive peak was discovered at 1419 and 1407 cm^−1^, which was attributed to C=C of the required octahydroquinazolines of compounds **4a** and **4b**, respectively [28]. In compound **4b**, there is also a C=S peak coupled with a weak symmetric N–H bending at 813 cm^−1^ [29].

###### Raman Spectroscopy

**Figure 2:**
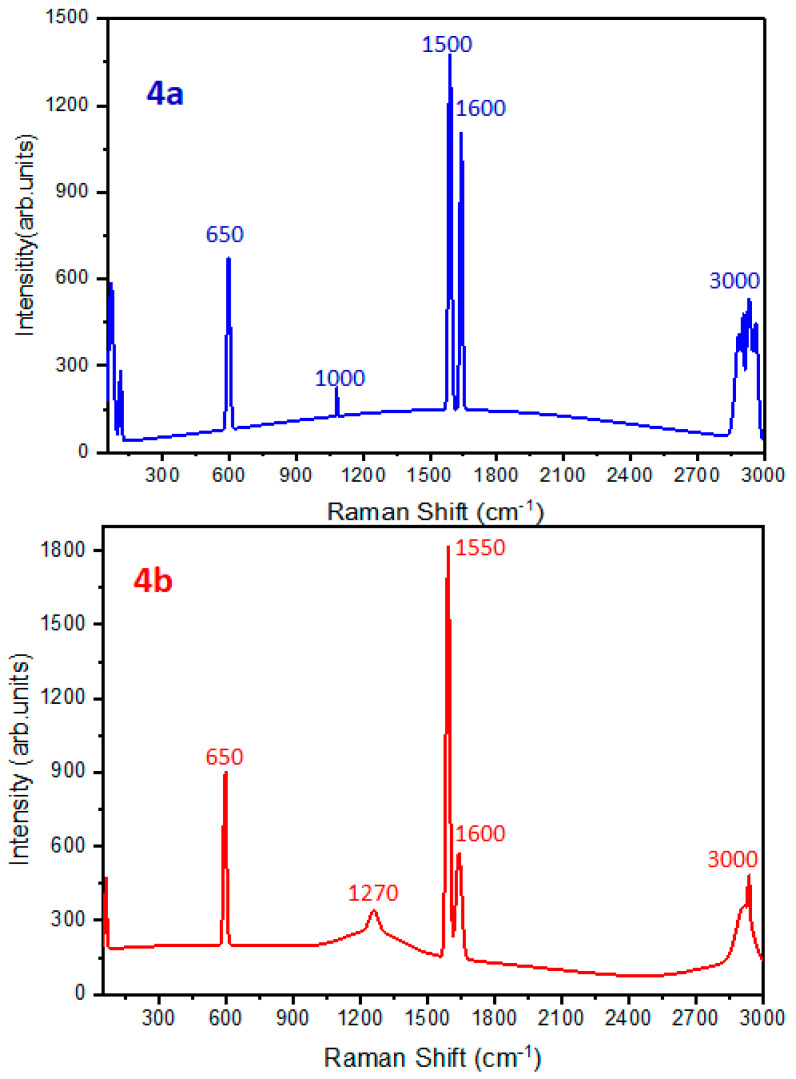
Raman spectra of compounds **4a** and **4b**.

The Raman spectra of compounds **4a** and **4b** showed a band at 1600 cm^−1^ attributed to C=O functional groups and a band around or above 3000 cm^−1^ attributed to both –NH- and phenyl OH groups, and these results were confirmed by the literature [30] as in Figure 2. There are many small peaks in the Raman spectra between 1000 and 1500 cm^−1^. In the Raman spectra, the in-plane distortion of the OH band is ascribed to 1450 cm^−1^. The stretching mode of the hydroxyl groups with respect to the phenyl moiety arises around 1270 cm^−1^ in the Raman spectra, as expected [31]. In the Raman spectra of both of the compounds discussed, the unique C=C band was detected around 1550 cm^−1^ [31]. In addition, another strong band at 650 cm^−1^ was identified in both the Raman spectra and must be assigned to the C=O and C=S compounds, respectively, for **4a** and **4b**.

##### Spectroscopic Results of Novel Compounds

*4-(3-Hydroxy-2-methoxyphenyl)-7,7-dimethyl-4,6,7,8-tetrahydroquinazoline-2,5(1H,3H)-dione* (**4a**); creamy white powder (Yield = 92%; m.p. (°C) = 241–242); FT-IR (cm^−1^, ATR); 3477, 3363 (2NH), 3191(OH), 1683 (C=O, ring),1593 (CONH), 1539 (C=O, urea), 1419 (C=C); ^1^H NMR (CDCl_3_, 400 MHz): δ 10.37 (s, 1H, OH, C-3′), 6.92–6.88(t, 1H, *J* = 15.80 Hz, Ar-H, C-4′), 6.80–6.78(d, 1H, *J* = 7.96 Hz, Ar-H, C-5′), 6.53–6.51(d, 1H, *J* = 7.56 Hz, Ar-H, C-6′), 5.03 (1H, s, CH, H-4), 3.78 (s, 3H, OCH_3_, C-2′), 3.35 (s, 2H, CH_2_, H-6), 2.21–2.05 (m, 2H, CH_2_, H-8), 1.04–0.88 (m, 6H, 2CH_3_, H-9, H-10); ^13^C NMR (CDCl_3_, 100 MHz): δ 196.24 (C=O, C-5), 165.88 (NC=O, C-2), 164.09 (NC=C, C-**8a**), 142.36 (OCH_3_, C-2′) 135.92 (C-OH, C-3′), 120.50 (ArC, C-6′), 115.04 (ArC, C-5′), 113.44 (ArC, C-4′), 106.17 (C=C, C-**4a**), 51.33 (1C, OCH_3_, C-3′’), 45.90 (1C, CH_2_, C-6), 45.22 (1C, CH, C-4), 38.45 (1C, CH_2_, C-8), 36.83 (1C, CH, C-7), 27.59 (1C, CH_3_, C-9), 26.19 (1C, CH_3_, C-10); ESI/MS *m/z* 314.3 [M − 2]^+^, 316.3 [M]^+^; Anal. Calcd for C_17_H_20_N_2_O_4_: C, 70.56; H, 6.37, N, 8.86. Found: C, 71.18, H, 6.69; N 9.33.

*4-(3-Hydroxy-2-methoxyphenyl)-7,7-dimethyl-2-thioxo-2,3,4,6,7,8-hexahydroquinazolin-5(1H)-one* (**4b**): White powder (Yield = 90%; m.p. (°C) = 235–236); FT-IR (cm^−1^, ATR); 3390, 3380 (2NH), 3305 (OH), 1685 (C=O, ring), 1595 (CONH), 1535 (C=O, urea), 1407 (C=C), 813 (C=S, thiourea); ^1^H NMR (CDCl_3_, 400 MHz): δ 10.37 (s, 1H, OH, C-3′), 6.90 (s, 1H, Ar-H, C-4′), 6.80–6.78 (d, 1H, *J* = 8.04 Hz, Ar-H, C-5′), 6.53–6.51 (d, 1H, *J* = 7.20 Hz, Ar-H, C-6′), 5.03 (1H, s, CH, H-4), 3.78 (s, 3H, OCH_3_, C-2′), 3.37 (s, 2H, CH_2_, H-6), 2.21 (s, 2H, CH_2_, H-8), 1.04–0.88 (m, 6H, 2CH_3_, H-9, H-10); ^13^C NMR (CDCl_3_, 100 MHz): δ 196.28 (C=O, C-5), 170.36 (NC=S, C-2), 165.03 (NC=C, C-**8a**), 147.14 (1C, Ar- OCH_3_, C-2′), 139.57 (1C, Ar-OH, C-3′), 126.68 (ArC, C-6′), 124.22 (ArC, C-5′), 120.13 (ArC, C-4′), 111.03 (C=C, C-4a), 55.93 (1C, OCH_3_, C-2′’), 50.90 (1C, CH2, C-6), 43.50 (1C, CH, C-4), 32.09 (1C, CH_2_, C-8), 29.61 (1C, CH, C-7), 26.70 (1C, CH_3_, C-9), 19.01 (1C, CH_3_, C-10); ESI/MS *m*/*z* 332.1 [M]^+^, 333.2 [M + 1]^+^; Anal. Calcd for C_17_H_20_N_2_O_3_S: C, 61.42; H, 6.06, N, 8.43. Found: C, 61.26, H, 6.72; N 8.33.

##### NMR Spectrum

In the ^1^H NMR spectra, the formation of the octahydroquinazolinone skeleton was clearly confirmed by the fact that the H4 methine proton of compounds **4a** and **4b** appeared at δ 5.03. One unreacted –OH of *o*-vanillin is available in both of the compounds at δ 10.37 ppm for H1, respectively.


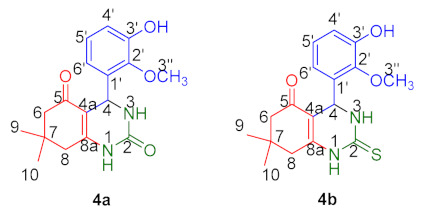


In the ^13^CNMR spectra of the compounds **4a** and **4b**, the most deshielded carbon atoms were located at C5 and C2. Both compounds have values of 196.2 ppm for the most deshielded carbon of C5. The second-most deshielded carbon at C2 belongs to >C=O in compound **4a,** which has δ 165.8 ppm. On the other hand, in compound **4b**, C2 of >C=S appeared in the spectra with an experimental value of 170.36 ppm. C=O and C=S resonances are slightly deshielded with higher chemical shifts, which may be due to intramolecular hydrogen bonding of compounds **4a** and **4b** and the electronegativity of oxygen and sulphur [32].

The most characteristic carbon at C4 resonates at δ 45.2 for compounds **4a** and **4b** at 43.50 ppm, respectively. Carbon 8a, for compounds **4a** and **4b,** resonates at δ 164.0 and 165.03 ppm, respectively. The signals for aromatic carbon and other primary, secondary, and tertiary carbon were displayed in the Supplementary Material (Figures S2 and S6).

### 2.2. Biological Evaluation

#### 2.2.1. Phospholipase (PLA_2_) Inhibitory Activity

**Figure 3:**
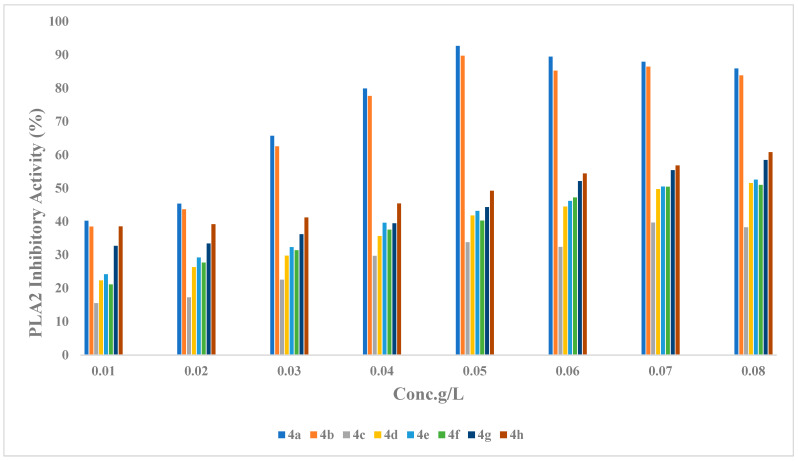
Inhibitory activity (%) of compounds **4a**–**h** against PLA2.

The phospholipase inhibitory activity of octahydroquinazolines **4a–h** has been detected at concentrations of 0.01 to 0.08 g/L, as shown in Figure 3. PLA2 inhibitory activity (inhibition) was found to be increasing with a corresponding increase in concentration for compounds **4a**–**h**. At a minimum concentration of 0.01 g/L, compounds **4a** and **4b** displayed 40.23% ± 2.41% and 38.46% ± 2.74%, while maximum activity for compounds **4a** and **4b** was exhibited at 92.86% ± 3.18% and 89.72% ± 3.66% at 0.05 g/L, as shown in Figure 3. For the rest of the compounds such as **4c, 4d, 4e, 4f, 4g,** and **4h** at each concentration, inhibitory activity against PLA2 was identified as insignificant. The results are somewhat in agreement with our previous work with the same enzyme, and the PLA_2_ inhibitory efficiencies of the current molecules are found to be even better than those of other established quinazolines or other moieties [18,23,25]. Oleanolic acid acts as a reference molecule and showed relatively less potency (72.43% ± 2.79%) as compared to compounds **4a** and **4b**, but more than other least active compounds (**4c, 4d, 4e, 4f, 4g,** and **4h**) at maximum concentration. By looking at the IC_50_ results of compounds **4a**–**h,** they represent moderate to high PLA2 activities, which are demonstrated in terms of ranges from 0.029–0.049 g/L. The highest significant activity belongs to compounds **4a** and **4b,** with IC_50_ values of 0.029 and 0.030 g/L, respectively (Table 1). The clinical significance of these compounds could be attributed to the fact that in many inflammatory conditions, PLA2 levels are elevated [33], and hence, they will be explored in such an ailment.

#### 2.2.2. Protease Activity

**Figure 4:**
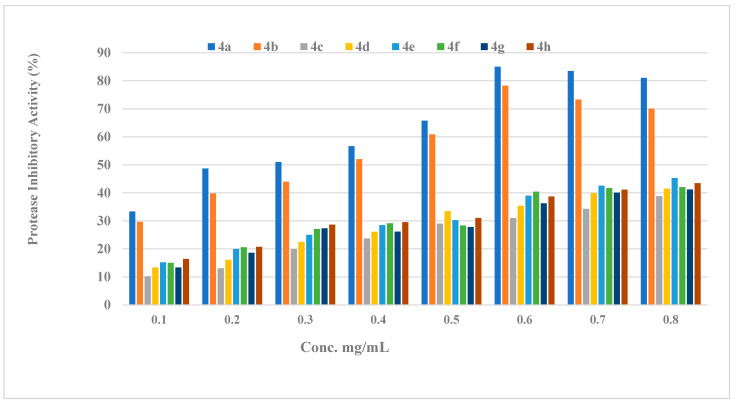
Antiprotease activity (%) of compounds **4a**–**h**.

The synthesized octahydroquinazolinones (**4a, 4b**) were screened against the protease K enzyme at a concentration of 0.1 to 0.8 mg/ mL, as shown in Figure 4. Protease inhibitory activity was exhibited in a dose-dependent manner. At all concentrations, **4a** and **4b** displayed better activity than the rest of the tested compounds (**4c, 4d, 4e, 4f, 4g,** and **4h**). Among these unsatisfactory compounds, the least protease inhibitory activity was observed at 0.1 mg/mL and was only 10 ± 2.14 for compound **4c** and 45 ± 1.89 for compound **4e** at 0.8 mg/mL. The maximum inhibition (85 ± 5.82) and (75 ± 4.66) was displayed at a concentration of 0.6 mg/mL by compounds **4a** and **4b,** respectively, which is better than some previous results, where protease inhibition was achieved at only 73.33% [18,24]. A protease inhibitor cocktail (Sigma) was used as the positive control, and the inhibitory potential was found to be close to maximum inhibition (83 ± 2.73). IC_50_ results for compounds **4a**–**h** for antiprotease activities demonstrated a range of 0.039–0.963 mg/L. The most promising candidates in terms of antiprotease activity were reported with IC_50_ values of 0.39 and 0.037 mg/L, respectively, for compounds **4a** and **4b**. Other compounds with IC_50_ values and their respective antiprotease activities showed moderate to good activity, as shown in Table 2. Regardless of the concentration, compound **4a** was the most promising candidate against phospholipase A2 (PLA2) and protease K enzymes under trial and could be presented as a prime anti-inflammatory agent.

#### 2.2.3. Structure Activity Relationship (SAR) of Compounds (**4a**–**h**)

The condensation of dimedone (**1**), urea/thiourea (**2**), and substituted aldehydes (**3a**–**h**) yielded a series of octahydroquinazolinone derivatives (**4a**–**h**) using 1% Pd-HPW/SiO_2_ as a catalyst. Only two of the synthesized compounds (**4a** and **4b**) showed satisfactory results in terms of inhibitory PLA2 (%) and antiprotease (%) activities. When the structure–activity relationship (SAR) was established among all tested compounds, no clear reason was revealed except a dose-dependent relationship among them. Due to the presence of electron-withdrawing (OCH_3_) and electron-donating (OH) functional groups on their respective phenyl moieties, two of the most promising compounds, **4a** and **4b**, perform as the best PLA2 and antiprotease agents. Due to the fact that their phenyl moieties include either electron-withdrawing (**4c, 4d, 4f, 4g**) or electron-donating (**4e, 4h**) groups, the remaining compounds do not exhibit any discernible enzymatic activity.

Additionally, two new compounds (**4a** and **4b**) have demonstrated amazing biological activity, leading us to conduct additional extended in silico experiments.

### 2.3. In Silico Studies

#### 2.3.1. Structure Elucidation of Compounds **4a** and **4b**

**Figure 5:**
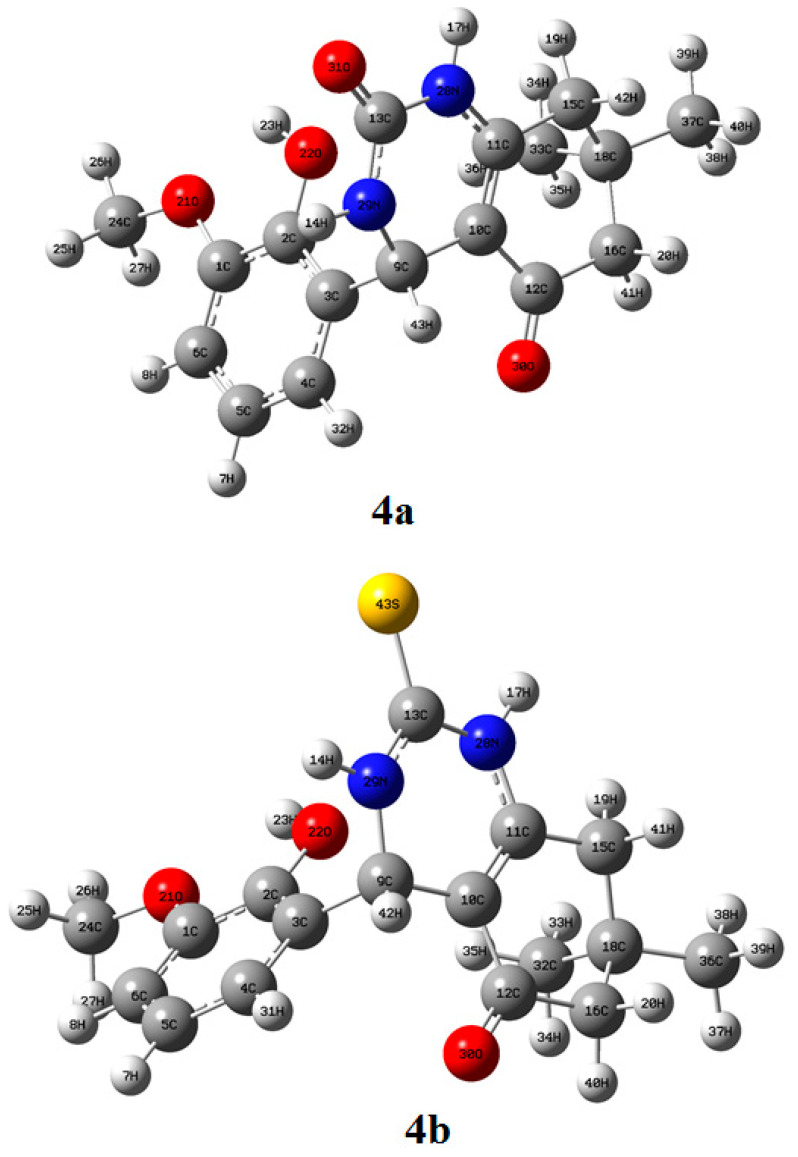
Optimized geometry of molecules **4a** and **4b** (B3LYP/cc-pVDZ).

Tables S1 and S2 show the Cartesian coordination and physical parameters of molecules **4a** and **4b,** respectively. Figure 5 shows the geometry of molecules **4a** and **4b**. From Table S2, notable bond lengths in molecule **4a** are C1–C2, C1–O21, C2–O22, C3–C9, C9–N29, C10–C12, C12–C16, C12–O30, C13–N28, C13–N29, C13–O31, H14–N29, H17–N28, O21–C24, O22–H23, C18–C33 and C18–C37 partaking 1.4117, 1.3772, 1.3663, 1.5304, 1.472, 1.4601, 1.5268, 1.2285, 1.403, 1.3669, 1.2206, 1.0146, 1.0126, 1.4195, 0.9728, 1.5421 and 1.5369 Å respectively; like **4b** are C1–C2, C1–O21, C2–O22, C3–C9, C9–N29, C12–O30, C13–N28, C13–N29, C13–S43, H14–N29, H17–N28, C18–C32, C18–C36, O21–C24, and O22–H23 having 1.4121, 1.376, 1.3648, 1.528, 1.4762, 1.2279, 1.3828, 1.3492, 1.6786, 1.0144, 1.0129, 1.542, 1.5368, 1.42 and 0.9729 Å in orderly, and bond angles are C10–C12–C16, C10–C12–O30, C16–C12–O30, N28–C13–N29, N28–C13–O31, N29–C13–O31, C1–O21–C24, C2–O22–H23, C11–N28–C13, C11–N28–H17, C13–N28–H17, C9–N29–C13, C9–N29–H14, and C13–N29–H14 by way of 117.284, 121.841, 120.832, 114.27, 120.873, 124.77, 118.223, 106.296, 124.353, 121.21, 114.422, 126.16, 116.947, and 112.61°, respectively; alike, **4b** are C2–C1–O21, C10–C12–C16, C10–1C2–O30, C16–C12–O30, N28–C13–N29, N28–C13–S43, N29–C13–S43, C1–O21–C24, C2–O22–H23, C11–N28–C13, C11–N28–C17, C13–N28–C17, C9–N29–C13, C9–N29–H14, and C13–N29–H14 having 113.149, 117.201, 121.663, 121.091, 115.152, 120.55, 124.252, 118.237, 106.468, 124.224, 120.987, 114.687, 126.834, 117.292, and 113.87° respectively of 3-methoxy-2-hydroxyphenyl, (sulphur) quinazoline-dione groups.

#### 2.3.2. Frontier Molecular Orbital (FMO) Analysis of Compounds **4a** and **4b**

**Figure 6:**
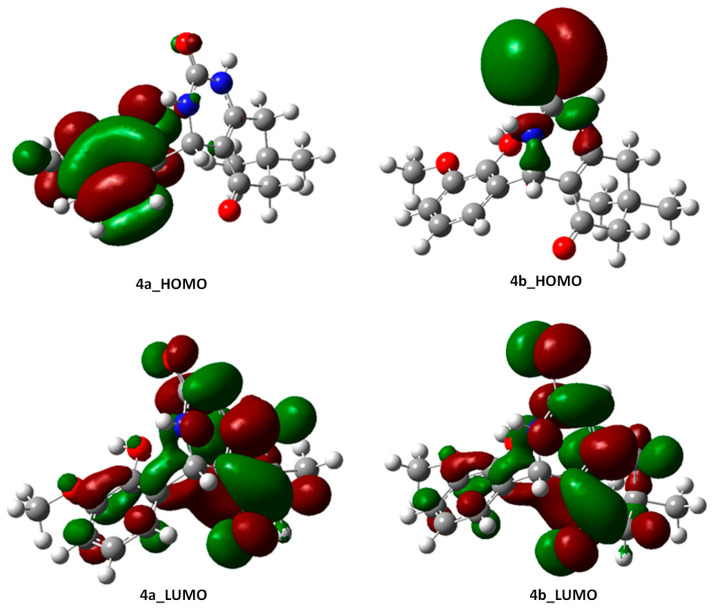
Frontier molecular orbitals of molecules **4a** and **4b**.

Table 3 shows the chemical reactivity and stability, and Figure 6 shows the frontier molecular orbitals of molecules **4a** and **4b** explained by some chemical descriptors. The electrophiles attack the sites of the highest occupied molecular orbital (HOMO) of molecule **4a**, spreading over the 3-methoxy-2-hydroxyphenyl group. In the case of molecule **4b,** it is spread over sulphur in the octahydroquinazolinone group, and the energies are −8.6269 and −7.5015 eV, respectively. The nucleophile attack sites of the lowest unoccupied molecular orbital (LUMO) of molecule **4a** are spread over the octahydroquinazolinone group, and those of molecule **4b** are spread over sulphur with the octahydroquinazolinone group, and the energies are −5.2689 and −5.3187 eV, respectively. The energy gap, which means electrons transfer from the valance band to the conduction band of molecules **4a** and **4b**, is −3.358 and −2.1828 eV, respectively, and this amount of energy is required for electron transitions. The smallest possible energies required for molecules 4a and 4b to form a cationic molecule are 8.62693 and 7.50153 eV, and these energies are known as ionization energies of corresponding molecules; similarly, the total amount of releasing energies required for molecules 4a and 4b to form an anionic molecule is 5.26894 and 5.31874 eV, which is also known as electron affinity of these molecules. All the molecular electrons can be stable at a particular energy point, which is mentioned as the hardness of molecules **4a** and **4b** at 1.67899 and 1.09139 eV, respectively; on the contrary, the energies 0.8395 and 0.5457 eV of molecules **4a** and **4b** electrons are unstable, which is called the softness of corresponding molecules. The total energies of molecules **4a** and **4b** are −6.9479 and −6.4101 eV, respectively; this is called the chemical potential energy of those molecules. When bonded electrons attract themselves, 6.94794 and 6.41013 eV of molecules 4a and 4b are required, indicating the electronegativity of the respective molecules. The amount of energies required for the addition of electrophiles to molecules 4a and 4b, respectively, is 14.3758 and 18.8245 eV, and this is known as the electrophilicity index; similarly, the amount of energies required for the addition of nucleophiles to corresponding molecules is 0.06956 and 0.05312 eV, and this is known as the nucleophilicity index. The energies 4.1337 and 6.48002 eV of molecules 4a and 4b are referred to as electron-accepting power, while the energies 11.0816 and 12.8901 eV of corresponding molecules are referred to as electron-donating power.

#### 2.3.3. Nature Bonding Orbital (NBO) Analysis of Molecules **4a** and **4b**

The inherent stability of the molecules, determined by intramolecular electron delocalizations, is very important to explain the nature of bonding orbitals via hyperconjugation. The ground state of nature of bonding orbital (NBO) calculations of molecules **4a** and **4b** were done using the NBO suite available within the Gaussian 09 software [34,35,36]. Table S3 depicts the nature of atomic orbitals with electron occupancies and energies of molecules **4a** and **4b**. In general, the decreasing order of atomic orbitals by the occupancies is core orbital > valence orbital > Rydberg orbital. Molecule **4a** has the following number of atoms C1, C2, C3, C4, C5, C6, H7, H8, C9, C10, C11, C12, C13, H14, C15, C16, H17, C18, H19, H20, O21, O22, H23, C24, H25, H26, H27, N28, N29, O30, O31, H32, C33, H34, H35, H36, C37, H38, H39, H40, H41, H42, and H43, and their valance atomic orbital numbers are 8, 22, 36, 46, 64, 78, 85, 90, 100, 112, 128, 144, 158, 165, 175, 191, 198, 208, 217, 222, 232, 248, 255, 263, 274, 279, 284, 292, 308, 322, 334, 345, 353, 364, 369, 374, 382, 393, 398, 403, 408, 413, and 418, respectively. They have the occupancies of electrons between 0.57354 and 1.80023 with the highest valance energies of electrons between 0.09804 and −0.0217; their type of atomic orbitals of hydrogen is Val(1S) with angular momentum is S, and carbon, nitrogen, and oxygen atoms having Val(2p) with their corresponding angular momentums are px/py/pz. 

Molecule **4b** has the following number of atoms C1, C2, C3, C4, C5, C6, H7, H8, C9, C10, C11, C12, C13, H14, C15, C16, H17, C18, H19, H20, O21, O22, H23, C24, H25, H26, H27, N28, N29, O30, H31, C32, H33, H34, H35, C36, H37, H38, H39, H40, H41, H42, and S43, their valance atomic orbital numbers are 8, 22, 36, 46, 64, 78, 85, 90, 100, 112, 128, 142, 158, 165, 173, 191, 198, 208, 217, 222, 232, 248, 255, 263, 274, 279, 284, 296, 308, 320, 331, 339, 350, 355, 360, 368, 379, 384, 389, 394, 399, 404, and 417, have the occupancies of electrons between 0.50103 and 1.81701 with highest valance energies of electrons between 0.09594 and −0.026. Their type of atomic orbitals of hydrogen is Val(1S) with angular momentum is S, and carbon, nitrogen oxygen and sulphur atoms having Val(2p) with their corresponding angular momentums are px/py/pz.

Table 4 shows the second-order perturbation theory analysis of the Fock matrix in the NBO basis of molecules **4a** and **4b**, explained by electron transfers from donor bonding/antibonding molecular orbitals to acceptor antibonding orbitals by absorbing/emitting some amount of energy. Molecule **4a** exhibits significant electron transfers from donor bonding and antibonding orbitals: BD (2) C1-C6, BD (2) C1–C6, BD (2) C2–C3, BD (2) C2–C3, BD (2) C4–C5, BD (2) C4–C5, BD (2) C10–C11, LP (2) O22, LP (1) N29, LP (1) O30, LP (2) O30, LP (2) O30, LP (1) O31, LP (2) O31, LP (2) O31, BD*(2) C1–C6, BD*(2) C1–C6 and BD*(2) C12–O30 to acceptor antibonding orbitals are BD*(2) C2–C3,BD*(2) C4–C5, BD*(2) C1–C6, BD*(2) C4–C5, BD*(2) C1–C6, BD*(2) C2–C3, BD*(2) C12–O30, BD*(2) C2–C3, BD*(2) C13–O31, RY*(1) C12, BD*(1) C10–C12, BD*(1) C12–C16, RY*(1) C13, BD*(1) C13–N28, BD*(1) C13–N29, BD*(2) C2–C3, BD*(2) C4–C5, and BD*(2) C10–C11 by the amount of energy used for absorbs/emits are 18.45, 17.84, 20.45, 20.36, 19.94, 19.06, 25.42, 27.61, 48.11, 15.00, 17.29, 19.62, 16.11, 26.39, 23.39, 301.87, 171.54, and 153.69 kcal/mol, respectively; similarly, molecule **4b** exhibits notable electron transfers from donor bonding/antibonding orbitals: BD (2) C1–C6, BD (2) C1–C6, BD (2) C2–C3, BD (2) C2–C3, BD (2) C4–C5, BD (2) C4–C5, BD (2) C10–C11, LP (2) O21, LP (2) O22, LP (1) N28, LP (1) N28, LP (1) N29, LP (1) N29, LP (1) O30, LP (2) O30, LP (2) O30, BD*(2) C1–C6, BD*(2) C1–C6, and BD*(2) C13–S43 to acceptor antibonding orbitals are BD*(2) C2–C3, BD*(2) C4–C5, BD*(2) C1–C6, BD*(2) C4–C5, BD*(2) C1–C6, BD*(2) C2–C3, BD*(2) C12–O30, BD*(2) C1–C6, BD*(2) C2–C3, BD*(2) C10–C11, BD*(2) C13–S43, BD*(1) C13–S43, BD*(2) C13–S43, RY*(1) C12, BD*(1) C10–C12, BD*(1) C12–C16, BD*(2) C2–C3, BD*(2) C4–C5 and BD*(1) C13–S43 by the amount of energy used for absorbs/emits are 18.65, 17.90, 20.08, 20.38, 19.89, 18.99, 24.62, 26.26, 28.02, 42.27, 22.72, 14.16, 24.23, 14.98, 17.49, 19.59, 351.67, 177.80, and 110.50 kcal/mol, respectively.

#### 2.3.4. Average Localized Ionization Energy (ALIE) of Molecules **4a** and **4b**

**Figure 7:**
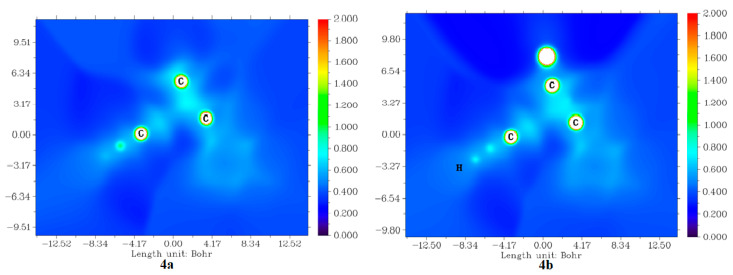
Average localizedionization energy of molecules **4a** and **4b**.

The average localized ionization energy (ALIE) study predicts the local ionization energy required for the electronic excitations, which is a wave function-based property, determined using the multi-wave function software with the help of the optimized geometry. Figure 7 represents the ALIE profile of the two compounds under study, which can be represented as a colored region from blue to red with scale values between 0.00 and 2.00, and the −12.50 to 12.50 Bohr^3^ volume range. Blueish-green indicates the delocalization of electrons in **4a** at 3-methoxy-2-hydroxylphenyl, oxygen, and nitrogen atoms in the azolin ring in quinazoline-dione groups, and in **4b** at 3-methoxy-2-hydroxylphenyl, sulphur, oxygen, and nitrogen atoms in the azolin ring in quinazoline-dione groups. The blue color indicates the sigma bond and the stable bond between atoms in the molecule. The sites are from protons and carbons and lone pairs of electrons in sulphur, oxygen, and nitrogen atoms.

#### 2.3.5. Molecular Electrostatic Potentials (MESP) from Electronic Charges and Nuclear Charges of Compounds **4a** and **4b**

**Figure 8:**
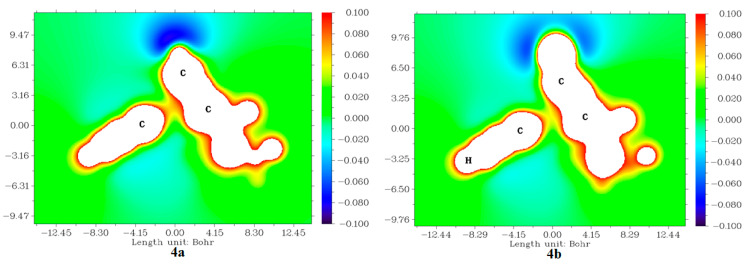
Molecular electrostatic potentials from electronic charges of molecules **4a** and **4b**.

Electrostatic potential can give an idea of the 3D charge distribution of the molecules, which helps to identify the possible electrophilic and nucleophilic centers. This potential can arise from electrons as well as nuclear charge. Figure 8 shows the MESP of the compounds due to electronic charges. The compounds show a color range from blue to red with scale values from −0.10 to 0.10 and from −12.45 to 12.45 in the Bohr^3^ range. Molecule **4a** has a blue color on all the nitrogen atoms of the octahydroquinazolinone group and the oxygen atoms of CH_3_O and OH in the 3-methoxy-2-hydroxylphenyl and carbonyl octahydroquinazolinone groups. These are electron-rich sites, so electrophiles can quickly attack. The red color is at carbon atoms which form a sigma bond with hydrogen atoms in the entire molecule, and they are electron-deficient sites; thus, nucleophiles can quickly attack these sites; and **4b**, which has the blue color, has all of the nitrogen atoms present at the octahydroquinazolinone group, oxygen atoms at methoxy-hydroxyl in 3-methoxy-2-hydroxylphenyl and carbonyl in octahydroquinazolinone groups, and sulphur in the octahydroquinazolinone group, these are electron-rich sites, so electrophiles can quickly attack these sites. The red color on all the carbon atoms forms a sigma bond with hydrogen atoms on the whole; these are electron-poor sites, so nucleophiles can quickly attack these sites.

#### 2.3.6. Molecular Docking

Molecular docking was performed to confirm the compound’s experimental biological activity and find the exact mechanism of action [37,38]. Structure–activity relationship (SAR) is an approach designed to find relationships between the chemical structure (or structurally related properties) and the biological activity (or target property) of the studied compounds. Pyrrole derivative compounds are potent inhibitors of human non-pancreatic secretory phospholipase A2 (hnps-PLA_2_) enzyme activity.

The molecular surface area as well as solvent accessibilities of protein (PDB ID: 1DB4) with molecules **4a** and **4b** are 2038.77 and 3179 (Å)^2^, while protein (PDB ID: 2PWB) with molecules **4a** and **4b** are 2355.89 and 2534.51 (Å)^2^, respectively. These solvent accessibilities will explain the surface area interactions between ligands and protein pockets (size and number of residues). Table 5 displays the docking score between proteins (PDB ID: 1DB4) and molecules 4a and 4b, which are 7.80 and 7.30 kcal/mol, respectively, and explains by modes the distributions from root-mean-square deviation from the upper lobe and the distributions from root-mean-square deviation from the lower lobe. Table 6 provides the details of the nature of the interaction between the drug and the protein.

**Figure 9:**
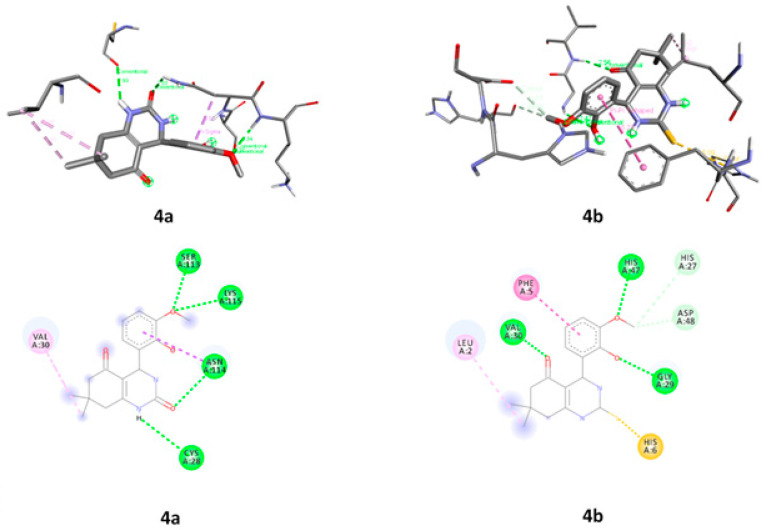
Interaction between molecules and protein 1DB4.

**Figure 10:**
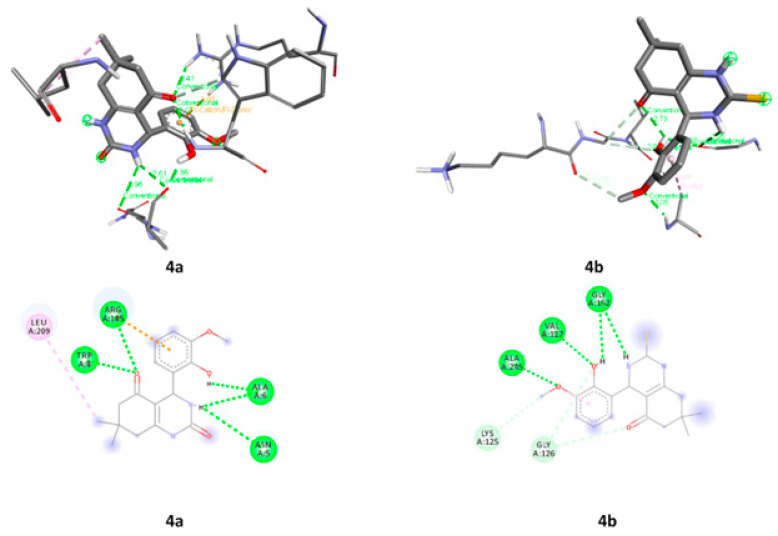
Interaction between molecules and protein 2PWB.

Figure 9 and Figure 10 show docking interactions between molecules **4a** and **4b** and proteins with PDB IDs 1DB4 and 2PWB, respectively.

Table S4 illustrates the non-bond and unsatisfied bonds of molecules **4a** and **4b** with proteins (PDB ID: 1DB4 and 2PWB), and also explains bond distances, categories, types, and chemistry. The protein PDB ID: 1DB4 with **4a** has four conventional hydrogen bonds, one pi–sigma bond, and two alkyl–alkyl bonds. In the same way, protein PDB ID: 1DB4 with **4b** has three conventional hydrogen bonds, two carbon–hydrogen bonds, one pi-sulfur bond, one pi–pi T-shaped bond, and an alkyl–alkyl bond, respectively. The protein PDB ID: 2PWB with **4a** has five conventional hydrogen bonds, two carbon–hydrogen bonds, one pi–cation; pi–donor hydrogen bond, and one alkyl–alkyl bond. Similarly, protein PDB ID: 2PWB with **4b** has five conventional hydrogen bonds, three carbon–hydrogen bonds, and one alkyl–alkyl bond, respectively. The unsatisfied sites within molecules **4a** and **4b** interact with proteins PDB ID: 1DB4 and 2PWB; hydrogen and sulphur are behaving as donors, and oxygen is an acceptor.

Among these results, at a zero relative mean standard deviation (rmsd) value for both the upper and lower bases, molecule **4a** has shown interactions with five different protein residues. In the same way, molecule **4b** has shown interactions with eight and five different protein residues.

**Figure 11:**
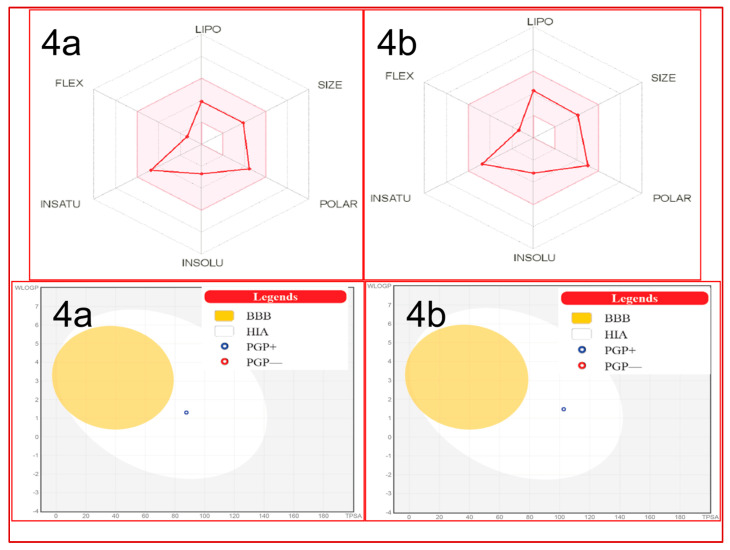
The bioavailability radar plots and boiled-egg graphs for the compounds **4a** and **4b** using Swiss ADME software.

The ADME parameters were predicted with the Swiss ADME software. None of the compounds violated Lipinski’s rule of five [39]. All the compounds (**4a**–**b**) were found to have sufficient lipophilicity for better absorption from the GIT, as shown in Figure 11. The ADME prediction profile is given in Table 7. The toxicity of the compounds was predicted using the ProTox II software, and both compounds were found to be non-toxic, with no hepatotoxicity, mutagenicity, carcinogenicity, immunotoxicity, or cytotoxicity predicted.

## 3. Materials and Methods

### 3.1. Chemistry

#### 3.1.1. Preparation of Pd-HPW/SiO_2_ Catalyst

Approximately, 2.96 g of 25% HPW/SiO_2_ powder and 0.02 M Pd(OAc)_2_ were added to a 200 mL beaker. The mixture was then poured with 14 mL of benzene and agitated gently for 1 h at room temperature on a magnetic stirrer, followed by progressive evaporation of the benzene in a rotary evaporator [26]. The catalyst must be calcined under vacuum at 150 °C/0.1 kPa after drying, and then reduced in an oven for 2 h by a hydrogen flow at 250 °C.

#### 3.1.2. General Procedure for the Synthesis of Octahydrquinazoline Derivatives (**4a**–**b**)

A combination of dimedone (**1**) (1 mmol), urea/thiourea (**2**) (1.5 mmol), and substituted aldehydes (**3**) (1 mmol) were agitated at 100 °C in a 100 mL round bottom flask in the presence of an optimal amount (0.1 g) of Pd-HPW/SiO_2_ catalyst in 10 mL of water [26]. The course of the reaction was continually monitored in a solvent system of ethyl acetate and acetone (3:7).

### 3.2. In Silico Studies

#### 3.2.1. DFT Studies

Compounds **4a** and **4b** were optimized using Gaussian-09 software [26,40] with the DFT- B3LYP [41,42,43,44,45,46], method, and cc-pVDZ [47,48,49,50,51] as a basis set, while the frontier molecular orbitals and nature bonding orbital analysis were performed at the same level. Compounds **4a** and **4b** have more than two reaction sites, for example, methoxy-, hydroxyl-, azolin-, and carbonyl/thiocarbonyl groups. Reaction sites of compounds **4a** and **4b** were calculated with the help of multi-wave function software by analyzing total electrostatic potential and average localized ionization energy [51].

#### 3.2.2. Molecular Docking

Biological activities were collected from PASS online [52,53,54], and corresponding protein activities were downloaded from the RCSB site [55] for protein data bank IDs: 1DB4 and 2PWB are macromolecules for human non-pancreatic secretory phospholipase A2 (hnps-PLA2 and protease K. The molecular docking analysis work was performed by using AutoDock Vina [56], Bio Discovery Studio, and package software [57]. We repeated the docking simulation multiple times to check whether the ligand binds to the same binding site, and in all cases, we found that the same binding site is preferred. Hence, we did not validate using another algorithm. Another reason for not validating the results is that the docking results are supported by experimental evidence.

##### ADME Studies

The ADME parameters for compounds **4a** and **4b** were predicted with the SwissADME server [58].

### 3.3. Biological Evaluation

#### 3.3.1. Inhibitory Activity of Phospholipase (PLA2) Enzyme

The test of PLA2 inhibitory activity was performed as discussed before by De Arajo and Radvanybm [59]. Commercially available phospholipase A2 procured from Sigma-Aldrich (Saint Louis, MO, USA) (P6534), was used in this assay. In 100 mL H_2_O, the substrate was made up of 3.5 mM lecithin, 3 mM NaTDC, 100 mM NaCl, 10 mM CaCl_2_, and 0.055 mM red phenol as a colorimetric indicator. Phosphate buffer was used to adjust the pH of the reaction mixture to 7.6. The sPLA2 protein was solubilized in 10% acetonitrile at a concentration of 0.01 to 0.08 g/L. For 20 min at room temperature, a volume of 10 L of these PLA2 solutions was incubated with a volume of 10 L containing 10 g of each compound. After that, 1 mL of PLA2 substrate was added, and the hydrolysis kinetics was monitored for 5 min by monitoring the optical density at 558 nm. The percentage of inhibition was estimated by comparing the results to a control experiment (devoid of compound). Oleanolic acid was used as a positive control in this experiment.

#### 3.3.2. Protease Inhibitory Activity

Protease K, obtained from commercially available sources (P2308, Sigma-Aldrich, Saint Louis, MO, USA), was used in this study. Briefly, protease tests were performed using Hammerstein casein as the substrate using the Kunitzcaseinolytic technique [60]. Protease inhibitory activities were measured under the same conditions, with the inhibitor (0.1 mg/mL) added to the reaction mixture and a 10 min pre-incubation at 37 °C. Following the remaining enzyme activity assay, 2 mL of 1% casein was added, and the mixture was allowed to stand for 30 min at 37 °C. The addition of 2.5 mL of a 5% TCA solution stopped the reaction. The absorbance of the reaction mixture was measured at 280 nm after centrifugation (12,000 rpm, 15 min). The amount of protease inhibitor that suppresses one unit of corresponding enzyme activity is known as a protease inhibitor unit. Along with the test, appropriate blanks for the enzyme, inhibitor, and substrate were run in parallel assays. A protease inhibitor cocktail (Sigma) was used as a positive control.

## 4. Conclusions

Herein, we reported the synthesis of a series of eight compounds and evaluated them for antiphospholipase (PLA2) and protease inhibitory activities. Among these two compounds, significant biological activities were observed against enzymes. Infrared spectroscopy, Raman, NMR, and mass spectroscopy techniques were used to characterize the novel molecules (**4a**, **4b**). Molecular docking simulation confirmed the observed biological activity of the two compounds, with **4a** having a higher docking score than **4b**: −7.80 and −7.30 kcal/mol with 1DB4 and −7.00 and −6.60 kcal/mol with 2PWB, which indicate strong interactions. The compound is found to contain delocalized electrons, which affect the stability of the molecule, which is further confirmed by the stability and reactivity parameters. From the FMO analysis, **4a** has a larger energy gap (∆E) than **4b,** whose values are −3.3580 and −2.1828 eV, respectively. The wave function properties, such as ALIE and MESP, of both electronic and nuclear charges are shown at the reactive sites. Because molecule **4b** has greater solvent accessibility for each protein than molecule **4a**, there are more interactions between molecules and protein residues.

## Figures and Tables

**Table 1:** IC_50_ results of phospholipase (PLA_2_) inhibitory activity.

Compound	IC_50_
**4a**	0.029 g/L (0.027–0.031, *n* = 3)
**4b**	0.030 g/L (0.028–0.031, *n* = 3)
**4c**	0.037 g/L (0.030–0.044, *n* = 3)
**4c**	0.041 g/L (0.037–0.046, *n* = 3)
**4e**	0.035 g/L (0.029–0.042, *n* = 3)
**4f**	0.028 g/L (0.011–0.044, *n* = 3)
**4g**	0.049 g/L (0.046–0.052, *n* = 3)
**4h**	0.048 g/L (0.044–0.053, *n* = 3)

**Table 2:** IC_50_ results of antiprotease activity.

Compound	IC_50_
**4a**	0.039 mg/L (0.031–0.046, *n* = 3)
**4b**	0.037 mg/L (0.032–0.042, *n* = 3)
**4c**	0.039 mg/L (0.023–0.042, *n* = 3)
**4c**	0.037 mg/L (0.033–0.040, *n* = 3)
**4e**	0.040 mg/L (0.032–0.049, *n* = 3)
**4f**	0.047 mg/L (0.011–0.067, *n* = 3)
**4g**	0.963 mg/L (0.315–2.94, *n* = 3)
**4h**	0.826 mg/L (0.382–1.78, *n* = 3)

**Table 3:** Frontier molecular orbital (FMO) properties of molecules **4a** and **4b**.

Parameters	4a		4b	
	eV	kcal/mol	eV	kcal/mol
HOMO	−8.6269	−198.42	−7.5015	−172.54
LUMO	−5.2689	−121.19	−5.3187	−122.33
Energy gap (∆E)	−3.3580	−77.234	−2.1828	−50.204
Ionization energy (I = ɛHOMO = −HOMO)	8.62693	198.419	7.50153	172.535
Electron affinity (A= ɛLUMO = −LUMO)	5.26894	121.186	5.31874	122.331
Global hardness (η = (I − A)/2)	1.67899	38.6168	1.09139	25.102
Global softness (S = 1/2η)	0.8395	19.3084	0.5457	12.551
Chemical potential (μ = −(I + A)/2)	−6.9479	−159.8	−6.4101	−147.43
Electronegativity (χ = −μ)	6.94794	159.803	6.41013	147.433
Electrophilicity index (ω = μ2/2η	14.3758	330.644	18.8245	432.963
Nucleophilicity index (N = 1/ω)	0.06956	1.59991	0.05312	1.22181
Electron accepting power (ω^+^ = A^2^/2(I − A))	4.1337	95.0751	6.48002	149.04
Electron donating power (ω^−^ = I^2^/2(I − A))	11.0816	254.8772	12.8901	296.473

**Table 4:** Second-order perturbation theory analysis of Fock matrix in NBO basis of molecules **4a** and **4b**.

NBOs	Donor NBO (i)	NBOs	Acceptor NBO (j)	E(2) kcal/mol	E(j)-E(i) a.u.	F(i,j) a.u.
**4a**						
3	BD (2) C1–C6	377	BD*(2) C2–C3	18.45	0.31	0.069
3	BD (2) C1–C6	382	BD*(2) C4–C5	17.84	0.31	0.067
6	BD (2) C2–C3	374	BD*(2) C1–C6	20.45	0.28	0.069
6	BD (2) C2–C3	382	BD*(2) C4–C5	20.36	0.30	0.07
11	BD (2) C4–C5	374	BD*(2) C1–C6	19.94	0.27	0.067
11	BD (2) C4–C5	377	BD*(2) C2–C3	19.06	0.28	0.067
20	BD (2) C10–C11	397	BD*(2) C12–O30	25.42	0.31	0.079
78	LP (2) O22	377	BD*(2) C2–C3	27.61	0.36	0.095
80	LP (1) N29	401	BD*(2) C13–O31	48.11	0.32	0.112
81	LP (1) O30	174	RY*(1) C12	15.00	1.30	0.125
82	LP (2) O30	392	BD*(1) C10–C12	17.29	0.75	0.103
82	LP (2) O30	395	BD*(1) C12–C16	19.62	0.65	0.102
83	LP (1) O31	183	RY*(1) C13	16.11	1.21	0.125
84	LP (2) O31	398	BD*(1) C13–N28	26.39	0.65	0.119
84	LP (2) O31	399	BD*(1) C13–N29	23.39	0.71	0.117
374	BD*(2) C1–C6	377	BD*(2) C2–C3	301.87	0.01	0.085
374	BD*(2) C1–C6	382	BD*(2) C4–C5	171.54	0.02	0.082
397	BD*(2) C12–O30	391	BD*(2) C10–C11	153.69	0.01	0.075
**4b**						
3	BD (2) C1–C6	381	BD*(2) C2–C3	18.65	0.30	0.069
3	BD (2) C1–C6	386	BD*(2) C4–C5	17.90	0.31	0.067
6	BD (2) C2–C3	378	BD*(2) C1–C6	20.08	0.28	0.068
6	BD (2) C2–C3	386	BD*(2) C4–C5	20.38	0.30	0.07
11	BD (2) C4–C5	378	BD*(2) C1–C6	19.89	0.27	0.067
11	BD (2) C4–C5	381	BD*(2) C2–C3	18.99	0.28	0.067
20	BD (2) C10–C11	401	BD*(2) C12–O30	24.62	0.31	0.078
80	LP (2) O21	378	BD*(2) C1–C6	26.26	0.36	0.093
82	LP (2) O22	381	BD*(2) C2–C3	28.02	0.36	0.096
83	LP (1) N28	395	BD*(2) C10–C11	42.27	0.31	0.106
83	LP (1) N28	405	BD*(2) C13–S43	22.72	0.35	0.08
84	LP (1) N29	404	BD*(1) C13–S43	14.16	0.43	0.071
84	LP (1) N29	405	BD*(2) C13–S43	24.23	0.34	0.081
85	LP (1) O30	178	RY*(1) C12	14.98	1.30	0.125
86	LP (2) O30	396	BD*(1) C10–C12	17.49	0.75	0.104
86	LP (2) O30	399	BD*(1) C12–C16	19.59	0.66	0.103
378	BD*(2) C1–C6	381	BD*(2) C2–C3	351.67	0.01	0.085
378	BD*(2) C1–C6	386	BD*(2) C4–C5	177.80	0.02	0.082
405	BD*(2) C13–S43	404	BD*(1) C13–S43	110.50	0.09	0.166

**Table 5:** Docking score of molecules with proteins.

Mode	Affinity kcal/mol	Distribution from Best Mode		Mode	Affinity kcal/mol	Distribution from Best Mode	
		rmsdl.b	rmsdu.b.			rmsdl.b	rmsdu.b.
**1DB4 vs. 4a**				**1DB4 vs. 4b**			
1	−7.80	0	0	1	−7.30	0	0
2	−6.80	1.806	2.167	2	−7.10	3.214	4.218
3	−6.70	9.86	11.519	3	−6.90	2.703	3.421
4	−6.60	2.494	5.878	4	−6.60	3.382	4.898
5	−6.50	1.87	2.322	5	−6.50	3.116	6.516
6	−6.20	2.225	5.731	6	−6.30	2.657	3.351
7	−6.00	9.6	11.567	7	−6.30	2.416	5.575
8	−5.90	3.292	4.99	8	−6.30	4.985	6.505
9	−5.90	2.104	2.917	9	−6.20	3.059	5.453
**Mode**	**Affinity kcal/mol**	**Distribution from Best Mode**		**Mode**	**Affinity kcal/mol**	**Distribution from Best Mode**	
		**rmsdl.b**	**rmsdu.b.**			**rmsdl.b**	**rmsdu.b.**
**2PWB vs. 4a**				**2PWB vs. 4b**			
1	−7.00	0	0	1	−6.60	0	0
2	−6.60	2.572	4.249	2	−6.50	3.067	4.547
3	−6.40	16.378	18.226	3	−6.30	4.012	7.627
4	−6.40	2.717	5.942	4	−6.30	2.764	6.511
5	−6.30	2.172	5.757	5	−6.20	17.163	19.589
6	−6.10	2.813	5.743	6	−6.00	23.322	25.106
7	−6.00	20.991	22.976	7	−5.90	2.767	4.068
8	−5.90	23.184	24.946	8	−5.80	27.71	29.173
9	−5.80	27.267	29.093	9	−5.70	29.691	30.831

**Table 6:** Indications the docking interactions between molecules **4a** and **4b** and proteins (PDB ID: 1DB4 and 2PWB).

Compounds	Proteins	Protein Residues
**4a**	1DB4	CYS A:28, VAL A:30, SER A:113, ASN A:114 and LYS A:115
**4b**		LEU A:2, PHE A:5, HIS A6, HIS A:27, GLY A:29, VAL A:30, HIS A:47 and ASP A:48
**4a**	2PWB	ASN A:5, ALA A:6, TRP A:8, ARG A:185 and LEU A:209
**4b**		LYS A:125, GLY A:126, VAL A:127, GLY A:152 AND ALA A:245

**Table 7:** ADME and toxicity prediction of compounds (**4a**–**b**).

Parameters	Compounds	
	4a	4b
Molecular weight	316.35	332.42
No. H-bond acceptors	4	3
No. H-bond donors	3	3
LogP_O/W_(iLOGP)	2.36	2.76
No. rotatable bonds	2	2
TPSA	87.66	102.68
Log K_P_ (skin permeation)	−7.28	−6.95
Lipinski’s rule violation	No	No
Bioavailability score	0.55	0.55
GI absorption	High	High
BBB permeation	No	No
Hepatotoxicity	−	−
Immunotoxicity	−	−
Mutagenicity	−	−
Cytotoxicity	−	−

(−) Showed the compound is devoid of any predicted toxicity.

## Data Availability

Data can be found in the published manuscript and its Supplementary Materials.

## Supplementary Materials

The following are available online at https://www.mdpi.com/article/10.3390/molecules28041944/s1, Figures S1–S4: 1H NMR, 13C NMR, mass and IR spectra of compound **4a**; Figures S5–S8: 1H NMR, 13C NMR, mass and IR spectra of compound **4b**; Table S1: Cartesian coordination of molecules **4a** and **4b**; Table S2: Physical parameters of molecules **4a** and **4b**; Table S3: Natural atomic orbital occupancies of molecules **4a** and **4b**; Table S4: Non-bond interactions of molecules (**4a** and **4b**) with proteins (PDB IDs: 1DB4 and 2PWB).

## References

[1] Sabir S., Alhazza M.I., Ibrahim A.A. (2016). A review on heterocyclic moieties and their applications. Catal. Sustain. Energy.

[2] Riadi Y., Alamri M.A., Geesi M.H., Anouar E.H., Ouerghi O., Alabbas A.B., Alossaimi M.A., Altharawi A., Dehbi O., Alqahtani S.M. (2021). Synthesis, characterization, biological evaluation and molecular docking of a new quinazolinone-based derivative as a potent dual inhibitor for VEGFR-2 and EGFR tyrosine kinases. J. Biomol. Struct. Dyn..

[3] Allah M.A.A.H., Balakit A.A., Salman H.I., Abdulridha A.A., Sert Y. (2022). New Heterocyclic Compound as Carbon Steel Corrosion Inhibitor in 1 M H_2_SO_4_, High Efficiency at Low Concentration: Experimental and Theoretical Studies. J. Adhes. Sci. Technol..

[4] Abdulridha A.A., Allah M.A.A.H., Makki S.Q., Sert Y., Salman H.E., Balakit A.A. (2020). Corrosion inhibition of carbon steel in 1 M H2SO4 using new Azo Schiff compound: Electrochemical, gravimetric, adsorption, surface and DFT studies. J. Mol. Liq..

[5] Balakit A.A., Makki S.Q., Sert Y., Ucun F., Alshammari M.B., Thordarson P., El-Hiti G.A. (2020). Synthesis, spectrophotometric and DFT studies of new Triazole Schiff bases as selective naked-eye sensors for acetate anion. Supramol. Chem..

[6] Gümüş M., Babacan N., Demir Y., Sert Y., Koca I., Gülçin I. (2021). Discovery of sulfadrug–pyrrole conjugates as carbonic anhydrase and acetylcholinesterase inhibitors. Arch. Pharm..

[7] Wan Y., Wu S., Zheng S., Liang E., Liu S., Yao X., Zhu Q. (2020). A series of octahydroquinazoline-5-ones as novel inhibitors against dengue virus. Eur. J. Med. Chem..

[8] Kidwai M., Bhatnagar D., Kumar R., Luthra P.M. (2010). Synthesis of 2-Oxo/Thioxooctahydroquinazolin-5-one Derivatives and Their Evaluation as Anticancer Agents. Chem. Pharm. Bull..

[9] Chandrika P.M., Yakaiah T., Rao A.R.R., Narsaiah B., Reddy N.C., Sridhar V., Rao J.V. (2008). Synthesis of novel 4,6-disubstituted quinazoline derivatives, their anti-inflammatory and anti-cancer activity (cytotoxic) against U937 leukemia cell lines. Eur. J. Med. Chem..

[10] Saleh E.A.M., Al Dawsari A.M., Husain K., Kutty I.H., Rai K. (2021). Synthesis, Antibacterial, and Antioxidant Evaluation of Novel Series of Condensed Thiazoloquinazoline with Pyrido, Pyrano, and Benzol Moieties as Five- and Six-Membered Heterocycle Derivatives. Molecules.

[11] Hassani Z., Islami M.R., Kalantari M. (2006). An efficient one-pot synthesis of octahydroquinazolinone derivatives using catalytic amount of H2SO4 in water. Bioorganic Med. Chem. Lett..

[12] Kuraitheerthakumaran A., Pazhamalai S., Manikandan H., Gopalakrishnan M. (2014). Rapid and efficient one-pot synthesis of octahydroquinazolinone derivatives using lanthanum oxide under solvent-free condition. J. Saudi Chem. Soc..

[13] Misono M. (2001). Unique acid catalysis of heteropoly compounds (heteropolyoxometalates) in the solid state. Chem. Commun..

[14] Furimsky E., Massoth F.E. (1993). Introduction. Catal. Today.

[15] Krayem N., Gargouri Y. (2020). Scorpion venom phospholipases A2: A minireview. Toxicon.

[16] Valentin E., Lambeau G. (2000). Increasing molecular diversity of secreted phospholipases A2 and their receptors and binding proteins. Biochim. Biophys. Acta (BBA) Mol. Cell Biol. Lipids.

[17] Murakami M., Kudo I. (2001). Diversity and regulatory functions of mammalian secretory phospholipase A2s. Adv. Immunol..

[18] El-Sayed N.N.E., Alafeefy A.M., Bakht M.A., Masand V.H., Aldalbahi A., Chen N., Fan C., Ben Bacha A. (2016). Synthesis, Antiphospholipase A2, Antiprotease, Antibacterial Evaluation and Molecular Docking Analysis of Certain Novel Hydrazones. Molecules.

[19] Burke J.E., Dennis E.A. (2009). Phospholipase A2 structure/function, mechanism, and signaling. J. Lipid Res..

[20] Rae D., Beechey-Newman N., Sumar N., Hermon-Taylor J., Porter J., Bennett D. (1994). Type 1 prophospholipase A2 propeptide in acute lung injury. Lancet.

[21] Peuravuori H.J., Funatomi H., Nevalainen T.J. (1993). Group I and Group II Phospholipases A2 in Serum in Uraemia. Clin. Chem. Lab. Med..

[22] Garcia-Carreno F., Navarrete M., Toro D. (1997). Classification of Proteases without Tears. Biochem. Educ..

[23] El-Sayed N., Almaneai N., Bacha A., El-Ashrey M., Al-Zaben M., Almarhoon Z. (2022). Biological Evaluation, Molecular Docking Analyses, and ADME Profiling of Certain New Quinazolinones as Anti-colorectal Agents. ACS Omega.

[24] Kirincich S.J., Xiang J., Green N., Tam S., Yang H., Shim J., Shen M., Clark J., McKew J. (2009). Benzhy-drylquinazolinediones: Novel cytosolic phospholipase A2α inhibitors with improved physicochemical properties. Bioorg. Med. Chem..

[25] El-Sayed N.N.E., Almaneai N.M., Ben Bacha A., Al-Obeed O., Ahmad R., Abdulla M., Alafeefy A.M. (2019). Synthesis and evaluation of anticancer, antiphospholipases, antiproteases, and antimetabolic syndrome activities of some 3*H*-quinazolin-4-one derivatives. J. Enzym. Inhib. Med. Chem..

[26] Bakht A., Alotaibi M., Alharthi A.I., Din I.U., Ali A., Ali A., Ahsan M.J. (2021). Pd-HPW/SiO_2_ Bi-Functional Catalyst: Sonochemical Synthesis, Characterization, and Effect on Octahydroquinazolinone Synthesis. Catalysts.

[27] Yarım M., Saraç S., Kılıç F., Erol K. (2003). Synthesis and in vitro calcium antagonist activity of 4-aryl-7,7-dimethyl/1,7,7-trimethyl-1,2,3,4,5,6,7,8-octahydroquinazoline-2,5-dione derivatives. II Farm..

[28] Le Parc R., Freitas V.T., Hermet P., Cojocariu A.M., Cattoën X., Wadepohl H., Maurin D., Tse C.H., Bartlett J.R., Ferreira R.A.S. (2019). Infrared and Raman spectroscopy of non-conventional hydrogen bonding between *N*,*N*′-disubstituted urea and thiourea groups: A combined experimental and theoretical investigation. Phys. Chem. Chem. Phys..

[29] Tiwari D., Fermin D.J., Chaudhuri T.K., Ray A. (2015). Solution Processed Bismuth Ferrite Thin Films for All-Oxide Solar Photovoltaics. J. Phys. Chem. C.

[30] Panicker C.Y., Varghese H.T., Ambujakshan K., Mathew S., Ganguli S., Nanda A.K., Van Alsenoy C. (2009). FT-IR and FT-Raman spectra and *ab initio* calculations of 3-{[(2-hydroxyphenyl) methylene]amino}-2-phenylquinazolin-4(3H)-one. J. Raman Spectrosc..

[31] Colthup N., Daly L., Wiberly S. (1990). Introduction to Infrared and Raman Spectroscopy.

[32] Khairul W.M., Isa M.I.N., Samsudin A.S., Adli H.K., Ghazali S.R. (2014). Conductive biodegradable film of N-octyloxyphenyl-N′-(4-methylbenzoyl)thiourea. Bull. Mater. Sci..

[33] Alafeefy A.M., Awaad A., Abdel-Aziz H., El-Meligy R.M., Zain M.E., Al-Outhman M.R., Ben Bacha A. (2014). Synthesis and biological evaluation of certain 3-substituted benzylideneamino-2-(4-nitrophenyl)quinazolin-4(3H)-one derivatives. J. Enzym. Inhib. Med. Chem..

[34] Thomas R., Pooventhiran T. (2022). Study of the dynamics of the interaction of glycine and GABA with water and ethanol using theoretical tools. J. Mol. Liq..

[35] Thomas R., Pooventhiran T., Bakht A., Alzahrani A.Y., Salem M.A. (2022). Study of interaction between different solvents and neurotransmitters dopamine, l-adrenaline, and l-noradrenaline using LED, QTAIM and AIMD. J. Mol. Liq..

[36] Thomas R., Pooventhiran T., Rai D.P. (2022). Comprehensive Quantum Mechanical Study of Structural Features, Reactivity, Molecular Properties and Wave Function-Based Characteristics of Capmatinib. Advanced Materials and Nano Systems: Theory and Experiment—Part 2.

[37] Pooventhiran T., Rajashekaraiah K., Murthy S., Joseph R., Thomas R. (2023). Study of the Electronic Properties of a Fluoropyrazolecarbonitrile Derivative and Enhancement of Spectral Properties on Adsorption with Fullerene. Biointerface Res. Appl. Chem..

[38] Anaikutti P., Selvaraj M., Prabhakaran J., Pooventhiran T., Jeyakumar T.C., Thomas R., Makam P. (2022). Indolyl-4H-chromenes: Multicomponent one-pot green synthesis, in vitro and in silico, anticancer and antioxidant studies. J. Mol. Struct..

[39] Lipinski C.A., Lombardo F., Dominy B.W., Feeney P.J. (2001). Experimental and computational approaches to estimate solubility and permeability in drug discovery and development settings. Adv. Drug Deliv. Rev..

[40] Frisch M., Trucks G., Schlegel H., Scuseria G., Robb M., Cheeseman J., Scalmani G., Barone V., Mennucci B., Petersson G. (2013). Gaussian 09 Revision D.01.

[41] Becke A.D. (1993). Density-functional thermochemistry. III. The role of exact exchange. J. Chem. Phys..

[42] Becke A.D. (1993). A new mixing of Hartree–Fock and local density-functional theories. J. Chem. Phys..

[43] Schmider H., Becke A. (2000). Chemical content of the kinetic energy density. J. Mol. Struct. THEOCHEM.

[44] Becke A.D. (1988). Density-functional exchange-energy approximation with correct asymptotic behavior. Phys. Rev. A.

[45] Becke A.D., Johnson E.R. (2005). A density-functional model of the dispersion interaction. J. Chem. Phys..

[46] Becke A.D. (2014). Perspective: Fifty years of density-functional theory in chemical physics. J. Chem. Phys..

[47] Frisch M.J., Pople J.A., Binkley J.S. (1984). Self-consistent molecular orbital methods 25. Supplementary functions for Gaussian basis sets. J. Chem. Phys..

[48] Longuet-Higgins H.C., Pople J.A. (1957). Electronic Spectral Shifts of Nonpolar Molecules in Nonpolar Solvents. J. Chem. Phys..

[49] Krishnan R., Binkley J.S., Seeger R., Pople J.A. (1980). Self-consistent molecular orbital methods. XX. A basis set for correlated wave functions. J. Chem. Phys..

[50] Rassolov V.A., Ratner M.A., Pople J.A., Redfern P.C., Curtiss L.A. (2001). 6-31G* basis set for third-row atoms. J. Comput. Chem..

[51] Lu T., Chen F. (2012). Multiwfn: A multifunctional wavefunction analyzer. J. Comput. Chem..

[52] Lagunin A., Stepanchikova A., Filimonov D., Poroikov V. (2000). PASS: Prediction of activity spectra for biologically active substances. Bioinformatics.

[53] Filimonov D., Lagunin A.A., Gloriozova T.A., Rudik A., Druzhilovskii D.S., Pogodin P.V., Poroikov V.V. (2014). Prediction of the Biological Activity Spectra of Organic Compounds Using the Pass Online Web Resource. Chem. Heterocycl. Compd..

[54] Mary Y.S., Resmi K., Kumar V.S., Thomas R., Sureshkumar B. (2019). Detailed quantum mechanical, molecular docking, QSAR prediction, photovoltaic light harvesting efficiency analysis of benzil and its halogenated analogues. Heliyon.

[55] Burley S.K., Berman H.M., Bhikadiya C., Bi C., Chen L., Di Costanzo L., Christie C., Dalenberg K., Duarte J.M., Dutta S. (2018). RCSB Protein Data Bank: Biological macromolecular structures enabling research and education in fundamental biology, biomedicine, biotechnology and energy. Nucleic Acids Res..

[56] Trott O., Olson A.J. (2010). AutoDock Vina: Improving the speed and accuracy of docking with a new scoring function, efficient optimization, and multithreading. J. Comput. Chem..

[57] Discovery Studio BIOVA (2017). Discovery Studio Vizualizer Client, V17.

[58] Daina A., Michielin O., Zoete V. (2017). SwissADME: A free web tool to evaluate pharmacokinetics, drug-likeness and medicinal chemistry friendliness of small molecules. Sci. Rep..

[59] De Araújo A.L., Radvanyi F. (1987). Determination of phospholipase A2 activity by a colorimetric assay using a pH indicator. Toxicon.

[60] Kunitz M. (1947). Crystalline soybean trypsin inhibitor. J. Gen. Physiol..

