# Supplementary material for: Synthesis and Biological Evaluation of Octahydroquinazolinones as Phospholipase A2, and Protease Inhibitors: Experimental and Theoretical Exploration
Source: Molecules. 2023 Feb 17;28(4):1944. doi: 10.3390/molecules28041944 (PMC9963251; doi:10.3390/molecules28041944)
Supplement: Supplementary file 1 [file molecules-28-01944-s001.zip › molecules-2198334-supplementary.pdf]

Synthesis and Biological Evaluation of Octahydro-quinazolinones as Phospholipase A2, and Proteanase Inhibitors: Experimental and Theoretical Exploration

Md Afroz Bakht [1,*], Thangaiyan Pooventhiran [2,3], Renjith Thomas [2], Mehnaz Kamal [4], Israf Ud Din [1], Najeeb-Ur-Rahman [5], Imtiaz Ali [6], Noushin Ajmal [7], and Mohamed Jawed Ahsan [8,*]

[1] Department of Chemistry, College of Science and Humanity Studies, Prince Sattam Bin Abdulaziz University, P.O. Box- 83, Al-Kharj 11942, Saudi Arabia
[2] Department of Chemistry, St Berchmans College (Autonomous), Mahatma Gandhi University, Changanassery, Kerala, India
[3] Department of Mechanical Engineering, University Centre for Research & Development, Chandigarh University, Gharuan, Mohali, Punjab, India
[4] Department of Pharmaceutical Chemistry, College of Pharmacy, Prince Sattam Bin Abdulaziz University, P.O. Box- 83, Al-Kharj 11942, Saudi Arabia
[5] Department of Pharmacology, College of Pharmacy, Prince Sattam Bin Abdulaziz University, P.O. Box- 83, Al-Kharj 11942, Saudi Arabia
[6] PreparatoryCollege, Prince Sattam Bin Abdulaziz University, P.O. Box- 83, Al-Kharj 11942, Saudi Arabia
[7] Department of Basic Science and Humanities, Pratap University, Jaipur, Rajasthan, India
[8] Department of Pharmaceutical Chemistry, Maharishi Arvind College of Pharmacy, Ambabari Circle, Jaipur, Rajasthan 302 039, India
* Correspondence: Correspondence: m.bakht@psau.edu.sa (M.A.B.); jawedpharma@gmail.com (M.J.A.)

SUPPLEMENTAL MATERIALS

Electronic Supplementary Information of Compounds 4a and 4b

Table S1. Cartesian coordination of molecules **4a** and **4b.**

Atom.	Atomic number	Coordinates (Angstroms)				Atom	Atomic number	Coordinates (Angstroms)		
		X	Y	Z				X	Y	Z
**4a**						**4b**				
1	6	-3.0555	-0.5885	-0.4612		1	6	-3.0017	-0.667	-0.4235
2	6	-1.8406	0.07533	-0.2103		2	6	-1.7851	0.0082	-0.1848
3	6	-1.3372	0.16573	1.08764		3	6	-1.3148	0.18542	1.12115
4	6	-2.0632	-0.436	2.12401		4	6	-2.0741	-0.344	2.17855
5	6	-3.2577	-1.0979	1.87765		5	6	-3.272	-1.013	1.94539
6	6	-3.766	-1.175	0.57756		6	6	-3.7491	-1.1753	0.63637
7	1	-3.802	-1.5606	2.69206		7	1	-3.8466	-1.4175	2.78056
8	1	-4.6994	-1.6891	0.38959		8	1	-4.6886	-1.698	0.4571
9	6	-0.0444	0.91867	1.40655		9	6	-0.0214	0.94982	1.41274
10	6	1.20258	0.29934	0.80897		10	6	1.2216	0.28876	0.8528
11	6	1.85585	0.89979	-0.2194		11	6	1.99599	0.83258	-0.3085
12	6	1.71923	-0.9262	1.40548		12	6	1.68444	-0.9428	1.48595

13	6	0.43947	2.89816	-0.0908		13	6	0.59496	2.70714	-0.1796
14	1	-0.8833	2.87931	1.39462		14	1	-0.8722	2.84	1.33053
15	6	3.07609	0.31575	-0.8801		15	6	3.10791	0.19175	-0.8209
16	6	2.98981	-1.5195	0.80691		16	6	2.93521	-1.5999	0.90736
17	1	1.88229	2.54781	-1.487		17	1	1.99306	2.36419	-1.5241
18	6	3.21193	-1.2102	-0.6867		18	6	3.17331	-1.3354	-0.5941
19	1	3.05568	0.55859	-1.9495		19	1	3.11961	0.41601	-1.9024
20	1	3.82733	-1.1169	1.39273		20	1	3.7938	-1.2094	1.48627
21	8	-3.4211	-0.5807	-1.7867		21	8	-3.3232	-0.7481	-1.7602
22	8	-1.1601	0.63276	-1.2541		22	8	-1.0708	0.48095	-1.2493
23	1	-1.6994	0.51944	-2.0491		23	1	-1.6003	0.2855	-2.0417
24	6	-4.6461	-1.2018	-2.1588		24	6	-4.5386	-1.3866	-2.1208
25	1	-5.4963	-0.7257	-1.66		25	1	-5.4105	-0.8703	-1.6819
26	1	-4.7357	-1.0688	-3.2355		26	1	-4.599	-1.3347	-3.2158
27	1	-4.6324	-2.2707	-1.923		27	1	-4.5471	-2.4446	-1.8044
28	7	1.44085	2.12074	-0.6869		28	7	1.47355	2.06209	-0.7092
29	7	-0.1166	2.33911	1.02003		29	7	-0.0757	2.35235	0.93408
30	8	1.19214	-1.4427	2.38439		30	8	1.12354	-1.4153	2.4716
31	8	0.16182	4.00174	-0.5272		31	1	-1.6961	-0.2353	3.19669
32	1	-1.664	-0.3936	3.13047		32	6	2.10349	-2.044	-1.4493
33	6	2.17492	-1.9558	-1.5503		33	1	2.3193	-1.9155	-2.5234
34	1	2.34397	-1.758	-2.6134		34	1	2.09316	-3.1256	-1.2381
35	1	2.25704	-3.0354	-1.3953		35	1	1.09426	-1.6486	-1.2594
36	1	1.15154	-1.659	-1.3114		36	6	4.56324	-1.8517	-0.9984
37	6	4.6237	-1.6512	-1.1059		37	1	4.63399	-2.9412	-0.8492
38	1	4.74779	-2.7296	-0.9717		38	1	4.7705	-1.6452	-2.0618
39	1	4.8103	-1.4241	-2.1606		39	1	5.35927	-1.3784	-0.4001
40	1	5.39259	-1.149	-0.5109		40	1	2.87697	-2.6774	1.12545
41	1	2.96978	-2.5966	0.99141		41	1	4.01114	0.66987	-0.3974
42	1	3.96065	0.8197	-0.4676		42	1	0.07955	0.96429	2.50945
43	1	0.06434	0.88058	2.4935		43	16	0.33308	4.18437	-1.0001

Table S2. Physical parameters of molecules **4a** and **4b.**

**Name**	**Definition**	**Values (in Å)**		**Name**	**Definition**	**Values (in °)**
**4a**						
R1	R(C1-C2)	1.4117		A1	A(C2-C1-C6)	120.547
R2	R(C1-C6)	1.393		A2	A(C2-C1-O21)	113.17
R3	R(C1-O21)	1.3772		A3	A(C6-C1-O21)	126.283
R4	R(C2-C3)	1.3993		A4	A(C1-C2-C3)	120.534

R5	R(C2-O22)	1.3663		A5	A(C1-C2-O22)	118.963
R6	R(C3-C4)	1.4053		A6	A(C3-C2-O22)	120.503
R7	R(C3-C9)	1.5304		A7	A(C2-C3-C4)	118.222
R8	R(C4-C5)	1.3918		A8	A(C2-C3-C9)	121.677
R9	R(C4-H32)	1.0915		A9	A(C4-C3-C9)	120.095
R10	R(C5-C6)	1.4027		A10	A(C3-C4-C5)	121.34
R11	R(C5-H7)	1.0915		A11	A(C3-C4-H32)	118.506
R12	R(C6-H8)	1.09		A12	A(C5-C4-H32)	120.146
R13	R(C9-C10)	1.5151		A13	A(C4-C5-C6)	120.313
R14	R(C9-N29)	1.472		A14	A(C4-C5-H7)	120.205
R15	R(C9-H43)	1.1014		A15	A(C6-C5-H7)	119.482
R16	R(C10-C11)	1.3629		A16	A(C1-C6-C5)	119.036
R17	R(C10-C12)	1.4601		A17	A(C1-C6-H8)	120.821
R18	R(C11-C15)	1.507		A18	A(C5-C6-H8)	120.142
R19	R(C11-N28)	1.3719		A19	A(C3-C9-C10)	113.891
R20	R(C12-C16)	1.5268		A20	A(C3-C9-N29)	112.216
R21	R(C12-O30)	1.2285		A21	A(C3-C9-H43)	105.887
R22	R(C13-N28)	1.403		A22	A(C10-C9-N29)	109.387
R23	R(C13-N29)	1.3669		A23	A(C10-C9-H43)	107.368
R24	R(C13-O31)	1.2206		A24	A(N29-C9-H43)	107.745
R25	R(H14-N29)	1.0146		A25	A(C9-C10-C11)	121.058
R26	R(C15-C18)	1.5453		A26	A(C9-C10-C12)	117.893
R27	R(C15-H19)	1.1046		A27	A(C11-C10-C12)	121.045
R28	R(C15-H42)	1.1062		A28	A(C10-C11-C15)	123.056
R29	R(C16-C18)	1.543		A29	A(C10-C11-N28)	120.415
R30	R(C16-H20)	1.1067		A30	A(C15-C11-N28)	116.511
R31	R(C16-H41)	1.1008		A31	A(C10-C12-C16)	117.284
R32	R(H17-N28)	1.0126		A32	A(C10-C12-O30)	121.841
R33	R(C18-C33)	1.5421		A33	A(C16-C12-O30)	120.832
R34	R(C18-C37)	1.5369		A34	A(N28-C13-N29)	114.27
R35	R(O21-C24)	1.4195		A35	A(N28-C13-O31)	120.873
R36	R(O22-H23)	0.9728		A36	A(N29-C13-O31)	124.77
R37	R(C24-H25)	1.1043		A37	A(C11-C15-H18)	113.267
R38	R(C24-H26)	1.0978		A38	A(C11-C15-H19)	109.036
R39	R(C24-H27)	1.1043		A39	A(C11-C15-H42)	108.336
R40	R(C33-H34)	1.1031		A40	A(C18-C15-H19)	110.117
R41	R(C33-H35)	1.1021		A41	A(C18-C15-H42)	109.656
R42	R(C33-H36)	1.1004		A42	A(H19-C15-H42)	106.167

R43	R(C37-H38)	1.102		A43	A(C12-C16-C18)	114.92
R44	R(C37-H39)	1.1029		A44	A(C12-C16-H20)	106.584
R45	R(C37-H40)	1.1025		A45	A(C12-C16-H41)	107.629
				A46	A(C18-C16-H20)	109.193
				A47	A(C18-C16-H41)	111.639
				A48	A(H20-C16-H41)	106.43
				A49	A(C15-C18-C16)	107.805
				A50	A(C15-C18-C33)	110.082
				A51	A(C15-C18-C37)	109.369
				A52	A(C16-C18-C33)	110.72
				A53	A(C16-C18-C37)	109.752
				A54	A(C33-C18-C37)	109.093
				A55	A(C1-O21-C24)	118.223
				A56	A(C2-O22-H23)	106.296
				A57	A(O21-C24-H25)	111.394
				A58	A(O21-C24-H26)	106.211
				A59	A(O21-C24-H27)	111.39
				A60	A(H25-C24-H26)	109.327
				A61	A(H25-C24-H27)	109.142
				A62	A(H26-C24-H27)	109.31
				A63	A(C11-N28-C13)	124.353
				A64	A(C11-N28-H17)	121.21
				A65	A(C13-N28-H17)	114.422
				A66	A(C9-N29-C13)	126.16
				A67	A(C9-N29-H14)	116.947
				A68	A(C13-N29-H14)	112.608
				A69	A(C18-C33-H34)	110.533
				A70	A(C18-C33-H35)	110.562
				A71	A(C18-C33-H36)	112.049
				A72	A(H34-C33-H35)	107.626
				A73	A(H34-C33-H36)	107.792
				A74	A(H35-C33-H36)	108.119
				A75	A(C18-C37-H38)	110.769
				A76	A(C18-C37-H39)	111.144
				A77	A(C18-C37-H40)	111.468
				A78	A(H38-C37-H39)	107.673
				A79	A(H38-C37-H40)	107.735
				A80	A(H39-C37-H40)	107.887

4b						
R1	R(C1-C2)	1.4121		A1	A(C2-C1-C6)	120.509
R2	R(C1-C6)	1.3929		A2	A(C2-C1-O21)	113.149
R3	R(C1-O21)	1.376		A3	A(C6-C1-O21)	126.342
R4	R(C2-C3)	1.3993		A4	A(C1-C2-C3)	120.438
R5	R(C2-O22)	1.3648		A5	A(C1-C2-O22)	119.116
R6	R(C3-C4)	1.4054		A6	A(C3-C2-O22)	120.446
R7	R(C3-C9)	1.528		A7	A(C2-C3-C4)	118.379
R8	R(C4-C5)	1.3914		A8	A(C2-C3-C9)	121.527
R9	R(C4-H31)	1.0915		A9	A(C4-C3-C9)	120.089
R10	R(C5-C6)	1.4029		A10	A(C3-C4-C5)	121.24
R11	R(C5-H7)	1.0914		A11	A(C3-C4-H31)	118.582
R12	R(C6-H8)	1.0899		A12	A(C5-C4-H31)	120.171
R13	R(C9-C10)	1.5136		A13	A(C4-C5-C6)	120.302
R14	R(C9-N29)	1.4762		A14	A(C4-C5-H7)	120.207
R15	R(C9-H42)	1.1013		A15	A(C6-C5-H7)	119.491
R16	R(C10-C11)	1.3612		A16	A(C1-C6-C5)	119.122
R17	R(C10-C12)	1.4615		A17	A(C1-C6-H8)	120.769
R18	R(C11-C15)	1.506		A18	A(C5-C6-H8)	120.108
R19	R(C11-N28)	1.3764		A19	A(C3-C9-C10)	114.334
R20	R(C12-C16)	1.5261		A20	A(C3-C9-N29)	111.608
R21	R(C12-O30)	1.2279		A21	A(C3-C9-H42)	106.122
R22	R(C13-N28)	1.3828		A22	A(C10-C9-N29)	109.156
R23	R(C13-N29)	1.3492		A23	A(C10-C9-H42)	107.745
R24	R(C13-S43)	1.6786		A24	A(N29-C9-H42)	107.552
R25	R(H14-N29)	1.0144		A25	A(C9-C10-C11)	121.022
R26	R(C15-C18)	1.5457		A26	A(C9-C10-C12)	117.921
R27	R(C15-H19)	1.1044		A27	A(C11-C10-C12)	121.05
R28	R(C15-H41)	1.1062		A28	A(C10-C11-C15)	123.224
R29	R(C16-C18)	1.5437		A29	A(C10-C11-N28)	120.283
R30	R(C16-H20)	1.1068		A30	A(C15-C11-N28)	116.466
R31	R(C16-H40)	1.1008		A31	A(C10-C12-C16)	117.201
R32	R(H17-N28)	1.0129		A32	A(C10-1C2-O30)	121.663
R33	R(C18-C32)	1.542		A33	A(C16-C12-O30)	121.091
R34	R(C18-C36)	1.5368		A34	A(N28-C13-N29)	115.152
R35	R(O21-C24)	1.42		A35	A(N28-C13-S43)	120.55
R36	R(O22-H23)	0.9729		A36	A(N29-C13-S43)	124.252
R37	R(C24-H25)	1.1042		A37	A(C11-C15-C18)	113.101
R38	R(C24-H26)	1.0977		A38	A(C11-C15-H19)	109.177

R39	R(C24-H27)	1.1042		A39	A(C11-C15-H41)	108.349
R40	R(C32-H33)	1.103		A40	A(C18-C15-H19)	110.092
R41	R(C32-H34)	1.102		A41	A(C18-C15-H41)	109.663
R42	R(C32-H35)	1.1004		A42	A(H19-C15-H41)	106.213
R43	R(C36-H37)	1.1019		A43	A(C12-C16-C18)	115.017
R44	R(C36-H38)	1.1028		A44	A(C12-C16-H20)	106.497
R45	R(C36-H39)	1.1025		A45	A(C12-C16-H40)	107.65
				A46	A(C18-C16-H20)	109.235
				A47	A(C18-C16-H40)	111.623
				A48	A(H20-C16-H40)	106.354
				A49	A(C15-C18-C16)	107.88
				A50	A(C15-C18-C32)	110.03
				A51	A(C15-C18-C36)	109.351
				A52	A(C16-C18-C32)	110.748
				A53	A(C16-C18-C36)	109.707
				A54	A(C32-C18-C36)	109.104
				A55	A(C1-O21-C24)	118.237
				A56	A(C2-O22-H23)	106.468
				A57	A(O21-C24-H25)	111.376
				A58	A(O21-C24-H26)	106.186
				A59	A(O21-C24-H27)	111.36
				A60	A(H25-C24-H26)	109.341
				A61	A(H25-C24-H27)	109.189
				A62	A(H26-C24-H27)	109.321
				A63	A(C11-N28-C13)	124.224
				A64	A(C11-N28-C17)	120.987
				A65	A(C13-N28-C17)	114.687
				A66	A(C9-N29-C13)	126.834
				A67	A(C9-N29-H14)	117.292
				A68	A(C13-N29-H14)	113.869
				A69	A(C18-C32-H33)	110.488
				A70	A(C18-C32-H34)	110.575
				A71	A(C18-C32-H35)	112.087
				A72	A(H33-C32-H34)	107.63
				A73	A(H33-C32-H35)	107.757
				A74	A(H34-C32-H35)	108.144
				A75	A(C18-C36-H37)	110.718
				A76	A(C18-C36-H38)	111.132
				A77	A(C18-C36-H39)	111.497

				A78	A(H37-C36-H38)	107.689
				A79	A(H37-C36-H39)	107.748
				A80	A(H38-C36-H39)	107.895

Table S3. Natural atomic orbital occupancies of molecules **4a** and **4b.**

NAO	Atom	No.	lang	Type (AO)	Occupancy	Energy		NAO	Atom	No.	lang	Type (AO)	Occupancy	Energy
**4a**														
1	C	1	S	Cor(1S)	1.99859	-10.119		**217**	**H**	**19**	**S**	**Val(1S)**	**0.76015**	**0.05047**
2	C	1	S	Val(2S)	0.82336	-0.1117		218	H	19	S	Ryd(2S)	0.00269	0.41442
3	C	1	S	Ryd(3S)	0.00191	1.03822		219	H	19	px	Ryd(2p)	0.0002	1.80053
4	C	1	px	Val(2p)	1.05449	-0.0722		220	H	19	py	Ryd(2p)	0.00012	1.74527
5	C	1	px	Ryd(3p)	0.00327	0.79017		221	H	19	pz	Ryd(2p)	0.00082	2.16275
6	C	1	py	Val(2p)	1.0551	-0.1039								
7	C	1	py	Ryd(3p)	0.00172	0.65241		**222**	**H**	**20**	**S**	**Val(1S)**	**0.75191**	**0.06611**
**8**	**C**	**1**	**pz**	**Val(2p)**	**0.77353**	**-0.0217**		223	H	20	S	Ryd(2S)	0.00204	0.4173
9	C	1	pz	Ryd(3p)	0.0093	0.78011		224	H	20	px	Ryd(2p)	0.00057	2.01463
10	C	1	dxy	Ryd(3d)	0.00066	1.88443		225	H	20	py	Ryd(2p)	0.00031	1.84115
11	C	1	dxz	Ryd(3d)	0.00154	1.91036		226	H	20	pz	Ryd(2p)	0.00035	1.89954
12	C	1	dyz	Ryd(3d)	0.00229	1.63171								
13	C	1	dx2y2	Ryd(3d)	0.00086	1.62539		227	O	21	S	Cor(1S)	1.99972	-18.986
14	C	1	dz2	Ryd(3d)	0.00192	2.05133		228	O	21	S	Val(2S)	1.59322	-0.8846
								229	O	21	S	Ryd(3S)	0.00038	2.11105
15	C	2	S	Cor(1S)	1.9985	-10.114		230	O	21	px	Val(2p)	1.61702	-0.3317
16	C	2	S	Val(2S)	0.81251	-0.0963		231	O	21	px	Ryd(3p)	0.0016	1.3739
17	C	2	S	Ryd(3S)	0.00183	1.12352		**232**	**O**	**21**	**py**	**Val(2p)**	**1.80023**	**-0.3272**
18	C	2	px	Val(2p)	0.9695	-0.0554		233	O	21	py	Ryd(3p)	0.00214	1.16324
19	C	2	px	Ryd(3p)	0.00651	0.81809		234	O	21	pz	Val(2p)	1.55893	-0.3366
20	C	2	py	Val(2p)	0.97896	-0.0931		235	O	21	pz	Ryd(3p)	0.00175	1.19214
21	C	2	py	Ryd(3p)	0.00298	0.66551		236	O	21	dxy	Ryd(3d)	0.00065	2.84831
**22**	**C**	**2**	**pz**	**Val(2p)**	**0.88862**	**-0.0341**		237	O	21	dxz	Ryd(3d)	0.00073	2.90696
23	C	2	pz	Ryd(3p)	0.0065	0.77358		238	O	21	dyz	Ryd(3d)	0.00068	2.77082

24	C	2	dxy	Ryd(3d)	0.00097	1.87664
25	C	2	dxz	Ryd(3d)	0.00189	1.97459
26	C	2	dyz	Ryd(3d)	0.00218	1.66421
27	C	2	dx2y2	Ryd(3d)	0.00137	1.63536
28	C	2	dz2	Ryd(3d)	0.00157	2.04515
29	C	3	S	Cor(1S)	1.99882	-10.047
30	C	3	S	Val(2S)	0.87806	-0.112
31	C	3	S	Ryd(3S)	0.00196	1.08551
32	C	3	px	Val(2p)	1.0594	-0.0583
33	C	3	px	Ryd(3p)	0.00666	0.97842
34	C	3	py	Val(2p)	1.05795	-0.0821
35	C	3	py	Ryd(3p)	0.0035	0.76841
**36**	**C**	**3**	**pz**	**Val(2p)**	**1.08588**	**-0.0436**
37	C	3	pz	Ryd(3p)	0.00458	0.98411
38	C	3	dxy	Ryd(3d)	0.0006	1.88066
39	C	3	dxz	Ryd(3d)	0.00073	1.98049
40	C	3	dyz	Ryd(3d)	0.00072	1.71894
41	C	3	dx2y2	Ryd(3d)	0.00073	1.65864
42	C	3	dz2	Ryd(3d)	0.00062	2.11151
43	C	4	S	Cor(1S)	1.99891	-10.041
44	C	4	S	Val(2S)	0.94635	-0.1283
45	C	4	S	Ryd(3S)	0.00113	1.06522
**46**	**C**	**4**	**px**	**Val(2p)**	**1.06201**	**-0.0414**
47	C	4	px	Ryd(3p)	0.00339	0.71964
48	C	4	py	Val(2p)	1.0221	-0.0747
49	C	4	py	Ryd(3p)	0.00181	0.61015

239	O	21	dx2y2	Ryd(3d)	0.00057	2.77096
240	O	21	dz2	Ryd(3d)	0.00125	3.09021
241	O	22	S	Cor(1S)	1.99977	-18.969
242	O	22	S	Val(2S)	1.65853	-0.9018
243	O	22	S	Ryd(3S)	0.00015	2.23055
244	O	22	px	Val(2p)	1.75205	-0.3262
245	O	22	px	Ryd(3p)	0.00139	1.24886
246	O	22	py	Val(2p)	1.83307	-0.3171
247	O	22	py	Ryd(3p)	0.00177	1.11072
**248**	**O**	**22**	**pz**	**Val(2p)**	**1.4462**	**-0.3031**
249	O	22	pz	Ryd(3p)	0.00229	1.23896
250	O	22	dxy	Ryd(3d)	0.0011	2.87453
251	O	22	dxz	Ryd(3d)	0.00129	3.22951
252	O	22	dyz	Ryd(3d)	0.00041	2.87488
253	O	22	dx2y2	Ryd(3d)	0.0012	2.80949
254	O	22	dz2	Ryd(3d)	0.00042	3.06286
**255**	**H**	**23**	**S**	**Val(1S)**	**0.50237**	**0.09804**
256	H	23	S	Ryd(2S)	0.00298	0.43606
257	H	23	px	Ryd(2p)	0.00154	1.93053
258	H	23	py	Ryd(2p)	0.00153	1.60089
259	H	23	pz	Ryd(2p)	0.00203	2.1695
260	C	24	S	Cor(1S)	1.99935	-10.153
261	C	24	S	Val(2S)	1.10755	-0.2852
262	C	24	S	Ryd(3S)	0.00157	1.07388
**263**	**C**	**24**	**px**	**Val(2p)**	**0.84494**	**-0.0781**

50	C	4	pz	Val(2p)	1.16945	-0.042		264	C	24	px	Ryd(3p)	0.00248	0.4755
51	C	4	pz	Ryd(3p)	0.00617	0.95081		265	C	24	py	Val(2p)	1.10606	-0.095
52	C	4	dxy	Ryd(3d)	0.00047	1.9024		266	C	24	py	Ryd(3p)	0.00102	0.50676
53	C	4	dxz	Ryd(3d)	0.00061	1.97508		267	C	24	pz	Val(2p)	1.17985	-0.1068
54	C	4	dyz	Ryd(3d)	0.00054	1.64605		268	C	24	pz	Ryd(3p)	0.003	0.68883
55	C	4	dx2y2	Ryd(3d)	0.00062	1.66916		269	C	24	dxy	Ryd(3d)	0.00156	1.72068
56	C	4	dz2	Ryd(3d)	0.00052	2.10824		270	C	24	dxz	Ryd(3d)	0.00101	1.70258
								271	C	24	dyz	Ryd(3d)	0.00053	1.65852
57	C	5	S	Cor(1S)	1.99899	-10.046		272	C	24	dx2y2	Ryd(3d)	0.00166	1.75829
58	C	5	S	Val(2S)	0.95143	-0.1367		273	C	24	dz2	Ryd(3d)	0.00099	1.90744
59	C	5	S	Ryd(3S)	0.00096	1.02193								
60	C	5	px	Val(2p)	1.0894	-0.0464		**274**	**H**	**25**	**S**	**Val(1S)**	**0.80514**	**0.03135**
61	C	5	px	Ryd(3p)	0.004	0.70442		275	H	25	S	Ryd(2S)	0.00275	0.36379
62	C	5	py	Val(2p)	1.0547	-0.0794		276	H	25	px	Ryd(2p)	0.00053	1.95401
63	C	5	py	Ryd(3p)	0.00225	0.62806		277	H	25	py	Ryd(2p)	0.00038	1.73045
**64**	**C**	**5**	**pz**	**Val(2p)**	**1.1217**	**-0.0359**		278	H	25	pz	Ryd(2p)	0.00033	1.73216
65	C	5	pz	Ryd(3p)	0.00515	0.84938								
66	C	5	dxy	Ryd(3d)	0.00045	1.84951		**279**	**H**	**26**	**S**	**Val(1S)**	**0.78549**	**0.04699**
67	C	5	dxz	Ryd(3d)	0.00062	2.05832		280	H	26	S	Ryd(2S)	0.00081	0.31147
68	C	5	dyz	Ryd(3d)	0.00058	1.69314		281	H	26	px	Ryd(2p)	0.0001	1.58937
69	C	5	dx2y2	Ryd(3d)	0.00057	1.62623		282	H	26	py	Ryd(2p)	0.00021	1.61184
70	C	5	dz2	Ryd(3d)	0.00053	1.98215		283	H	26	pz	Ryd(2p)	0.00088	2.16404
71	C	6	S	Cor(1S)	1.99892	-10.053		**284**	**H**	**27**	**S**	**Val(1S)**	**0.80538**	**0.03125**
72	C	6	S	Val(2S)	0.95672	-0.1475		285	H	27	S	Ryd(2S)	0.00273	0.36477
73	C	6	S	Ryd(3S)	0.00104	1.01945		286	H	27	px	Ryd(2p)	0.00014	1.59655
74	C	6	px	Val(2p)	1.15958	-0.0707		287	H	27	py	Ryd(2p)	0.00082	2.12171
75	C	6	px	Ryd(3p)	0.00417	0.86888		288	H	27	pz	Ryd(2p)	0.00029	1.69791

76	C	6	py	Val(2p)	1.09287	-0.0953
77	C	6	py	Ryd(3p)	0.00223	0.65911
**78**	**C**	**6**	**pz**	**Val(2p)**	**1.08071**	**-0.0443**
79	C	6	pz	Ryd(3p)	0.00315	0.69787
80	C	6	dxy	Ryd(3d)	0.00038	1.8815
81	C	6	dxz	Ryd(3d)	0.00067	1.91745
82	C	6	dyz	Ryd(3d)	0.00073	1.65491
83	C	6	dx2y2	Ryd(3d)	0.00052	1.62434
84	C	6	dz2	Ryd(3d)	0.00041	2.15611
**85**	**H**	**7**	**S**	**Val(1S)**	**0.76747**	**0.08321**
86	H	7	S	Ryd(2S)	0.00172	0.32591
87	H	7	px	Ryd(2p)	0.00034	1.93598
88	H	7	py	Ryd(2p)	0.00032	1.7071
89	H	7	pz	Ryd(2p)	0.00057	2.11209
**90**	**H**	**8**	**S**	**Val(1S)**	**0.76551**	**0.06814**
91	H	8	S	Ryd(2S)	0.00156	0.34978
92	H	8	px	Ryd(2p)	0.00072	2.06335
93	H	8	py	Ryd(2p)	0.0004	1.72437
94	H	8	pz	Ryd(2p)	0.0001	1.97006
95	C	9	S	Cor(1S)	1.99901	-10.103
96	C	9	S	Val(2S)	0.92516	-0.1802
97	C	9	S	Ryd(3S)	0.0014	1.51636
98	C	9	px	Val(2p)	1.04706	-0.0754
99	C	9	px	Ryd(3p)	0.00259	0.61256
**100**	**C**	**9**	**py**	**Val(2p)**	**0.84601**	**-0.0562**
289	N	28	S	Cor(1S)	1.99914	-14.206
290	N	28	S	Val(2S)	1.25807	-0.5369
291	N	28	S	Ryd(3S)	0.00114	1.74609
**292**	**N**	**28**	**px**	**Val(2p)**	**1.49551**	**-0.2603**
293	N	28	px	Ryd(3p)	0.00268	1.13186
294	N	28	py	Val(2p)	1.40506	-0.2578
295	N	28	py	Ryd(3p)	0.00212	1.13886
296	N	28	pz	Val(2p)	1.49293	-0.2572
297	N	28	pz	Ryd(3p)	0.00317	1.17796
298	N	28	dxy	Ryd(3d)	0.00056	2.38362
299	N	28	dxz	Ryd(3d)	0.00047	2.36145
300	N	28	dyz	Ryd(3d)	0.00055	2.42182
301	N	28	dx2y2	Ryd(3d)	0.00046	2.23195
302	N	28	dz2	Ryd(3d)	0.00036	2.21801
303	N	29	S	Cor(1S)	1.99929	-14.189
304	N	29	S	Val(2S)	1.28007	-0.5314
305	N	29	S	Ryd(3S)	0.0014	1.67056
306	N	29	px	Val(2p)	1.54452	-0.2333
307	N	29	px	Ryd(3p)	0.00363	1.14966
**308**	**N**	**29**	**py**	**Val(2p)**	**1.36784**	**-0.2253**
309	N	29	py	Ryd(3p)	0.00226	1.15948
310	N	29	pz	Val(2p)	1.48965	-0.2425
311	N	29	pz	Ryd(3p)	0.00188	1.05461
312	N	29	dxy	Ryd(3d)	0.00053	2.38361
313	N	29	dxz	Ryd(3d)	0.00052	2.32997
314	N	29	dyz	Ryd(3d)	0.00044	2.3491

101	C	9	py	Ryd(3p)	0.00637	0.65642
102	C	9	pz	Val(2p)	1.19258	-0.095
103	C	9	pz	Ryd(3p)	0.01156	0.9675
104	C	9	dxy	Ryd(3d)	0.00111	1.91198
105	C	9	dxz	Ryd(3d)	0.00056	1.89614
106	C	9	dyz	Ryd(3d)	0.00097	1.77473
107	C	9	dx2y2	Ryd(3d)	0.00176	1.87709
108	C	9	dz2	Ryd(3d)	0.00118	2.13667
109	C	10	S	Cor(1S)	1.99868	-10.037
110	C	10	S	Val(2S)	0.87691	-0.1086
111	C	10	S	Ryd(3S)	0.00243	1.19142
**112**	**C**	**10**	**px**	**Val(2p)**	**1.08759**	**-0.0686**
113	C	10	px	Ryd(3p)	0.00574	0.9277
114	C	10	py	Val(2p)	1.11947	-0.0742
115	C	10	py	Ryd(3p)	0.00467	0.95161
116	C	10	pz	Val(2p)	1.11298	-0.0725
117	C	10	pz	Ryd(3p)	0.00387	0.8705
118	C	10	dxy	Ryd(3d)	0.00122	1.60926
119	C	10	dxz	Ryd(3d)	0.00093	1.69192
120	C	10	dyz	Ryd(3d)	0.00094	1.69027
121	C	10	dx2y2	Ryd(3d)	0.00055	1.49286
122	C	10	dz2	Ryd(3d)	0.00112	1.44982
123	C	11	S	Cor(1S)	1.9988	-10.109
124	C	11	S	Val(2S)	0.81821	-0.1083
125	C	11	S	Ryd(3S)	0.00157	0.98885
126	C	11	px	Val(2p)	0.995	-0.0759

315	N	29	dx2y2	Ryd(3d)	0.00041	2.3334
316	N	29	dz2	Ryd(3d)	0.00086	2.2921
317	O	30	S	Cor(1S)	1.9998	-18.846
318	O	30	S	Val(2S)	1.69244	-0.8618
319	O	30	S	Ryd(3S)	0.00045	2.1155
320	O	30	px	Val(2p)	1.65299	-0.2391
321	O	30	px	Ryd(3p)	0.00169	1.18759
**322**	**O**	**30**	**py**	**Val(2p)**	**1.70538**	**-0.2381**
323	O	30	py	Ryd(3p)	0.0019	1.18927
324	O	30	pz	Val(2p)	1.53272	-0.2646
325	O	30	pz	Ryd(3p)	0.00059	1.06853
326	O	30	dxy	Ryd(3d)	0.00059	2.81898
327	O	30	dxz	Ryd(3d)	0.00098	3.07582
328	O	30	dyz	Ryd(3d)	0.00099	3.02434
329	O	30	dx2y2	Ryd(3d)	0.0005	2.80708
330	O	30	dz2	Ryd(3d)	0.00157	2.98672
331	O	31	S	Cor(1S)	1.9998	-18.845
332	O	31	S	Val(2S)	1.70036	-0.8586
333	O	31	S	Ryd(3S)	0.00076	2.09403
**334**	**O**	**31**	**px**	**Val(2p)**	**1.68446**	**-0.228**
335	O	31	px	Ryd(3p)	0.00123	1.14859
336	O	31	py	Val(2p)	1.57803	-0.2713
337	O	31	py	Ryd(3p)	0.0007	1.06204
338	O	31	pz	Val(2p)	1.68007	-0.2356
339	O	31	pz	Ryd(3p)	0.00093	1.14365
340	O	31	dxy	Ryd(3d)	0.00133	2.9699

127	C	11	px	Ryd(3p)	0.00455	0.88745		341	O	31	dxz	Ryd(3d)	0.00042	2.74939
**128**	**C**	**11**	**py**	**Val(2p)**	**0.88246**	**-0.0452**		342	O	31	dyz	Ryd(3d)	0.00129	3.08724
129	C	11	py	Ryd(3p)	0.00581	0.76111		343	O	31	dx2y2	Ryd(3d)	0.00122	3.02091
130	C	11	pz	Val(2p)	0.98807	-0.0751		344	O	31	dz2	Ryd(3d)	0.00083	2.84261
131	C	11	pz	Ryd(3p)	0.00378	0.73304								
132	C	11	dxy	Ryd(3d)	0.00107	1.90179		**345**	**H**	**32**	**S**	**Val(1S)**	**0.76183**	**0.08911**
133	C	11	dxz	Ryd(3d)	0.00083	2.05067		346	H	32	S	Ryd(2S)	0.00225	0.35609
134	C	11	dyz	Ryd(3d)	0.00086	2.00288		347	H	32	px	Ryd(2p)	0.00024	1.92784
135	C	11	dx2y2	Ryd(3d)	0.0014	1.86977		348	H	32	py	Ryd(2p)	0.00019	1.68567
136	C	11	dz2	Ryd(3d)	0.0013	1.8084		349	H	32	pz	Ryd(2p)	0.00078	2.2039
137	C	12	S	Cor(1S)	1.99905	-10.127		350	C	33	S	Cor(1S)	1.99927	-10.052
138	C	12	S	Val(2S)	0.80859	-0.107		351	C	33	S	Val(2S)	1.09594	-0.2458
139	C	12	S	Ryd(3S)	0.00536	1.00793		352	C	33	S	Ryd(3S)	0.00111	1.23157
140	C	12	px	Val(2p)	0.88247	-0.0386		**353**	**C**	**33**	**px**	**Val(2p)**	**1.16596**	**-0.0704**
141	C	12	px	Ryd(3p)	0.00747	0.78174		354	C	33	px	Ryd(3p)	0.00287	0.6071
142	C	12	py	Val(2p)	0.90805	-0.0312		355	C	33	py	Val(2p)	1.197	-0.0761
143	C	12	py	Ryd(3p)	0.0068	0.69832		356	C	33	py	Ryd(3p)	0.00325	0.6284
**144**	**C**	**12**	**pz**	**Val(2p)**	**0.78882**	**-0.0217**		357	C	33	pz	Val(2p)	1.17504	-0.075
145	C	12	pz	Ryd(3p)	0.01551	0.6737		358	C	33	pz	Ryd(3p)	0.00298	0.61349
146	C	12	dxy	Ryd(3d)	0.00165	1.98706		359	C	33	dxy	Ryd(3d)	0.00034	1.77222
147	C	12	dxz	Ryd(3d)	0.00206	1.91452		360	C	33	dxz	Ryd(3d)	0.00033	1.74977
148	C	12	dyz	Ryd(3d)	0.00176	1.97354		361	C	33	dyz	Ryd(3d)	0.00019	1.71682
149	C	12	dx2y2	Ryd(3d)	0.00184	1.8067		362	C	33	dx2y2	Ryd(3d)	0.00061	1.96778
150	C	12	dz2	Ryd(3d)	0.00318	1.79372		363	C	33	dz2	Ryd(3d)	0.00067	1.98149
151	C	13	S	Cor(1S)	1.9993	-10.191		**364**	**H**	**34**	**S**	**Val(1S)**	**0.78002**	**0.0648**
152	C	13	S	Val(2S)	0.68872	-0.0718		365	H	34	S	Ryd(2S)	0.00153	0.4009

153	C	13	S	Ryd(3S)	0.00514	0.67377
154	C	13	px	Val(2p)	0.8029	-0.0463
155	C	13	px	Ryd(3p)	0.00579	0.54941
156	C	13	py	Val(2p)	0.75832	0.0036
157	C	13	py	Ryd(3p)	0.01656	0.59942
**158**	**C**	**13**	**pz**	**Val(2p)**	**0.79981**	**-0.0214**
159	C	13	pz	Ryd(3p)	0.0097	0.54288
160	C	13	dxy	Ryd(3d)	0.00423	1.80426
161	C	13	dxz	Ryd(3d)	0.00201	1.85235
162	C	13	dyz	Ryd(3d)	0.00331	1.89795
163	C	13	dx2y2	Ryd(3d)	0.00287	1.72381
164	C	13	dz2	Ryd(3d)	0.00382	1.68693
**165**	**H**	**14**	**S**	**Val(1S)**	**0.57998**	**0.10051**
166	H	14	S	Ryd(2S)	0.00333	0.44529
167	H	14	px	Ryd(2p)	0.00121	2.00432
168	H	14	py	Ryd(2p)	0.00069	1.96042
169	H	14	pz	Ryd(2p)	0.00057	1.79695
170	C	15	S	Cor(1S)	1.99907	-10.071
171	C	15	S	Val(2S)	1.01849	-0.2342
172	C	15	S	Ryd(3S)	0.00165	1.46438
173	C	15	px	Val(2p)	1.13357	-0.0956
174	C	15	px	Ryd(3p)	0.00426	0.69168
**175**	**C**	**15**	**py**	**Val(2p)**	**1.09162**	**-0.0953**
176	C	15	py	Ryd(3p)	0.00392	0.59038
177	C	15	pz	Val(2p)	1.20628	-0.1011
178	C	15	pz	Ryd(3p)	0.00521	0.77501
366	H	34	px	Ryd(2p)	0.00018	1.71901
367	H	34	py	Ryd(2p)	0.00021	1.71498
368	H	34	pz	Ryd(2p)	0.00077	2.17172
**369**	**H**	**35**	**S**	**Val(1S)**	**0.77274**	**0.07132**
370	H	35	S	Ryd(2S)	0.00142	0.40027
371	H	35	px	Ryd(2p)	0.00017	1.72079
372	H	35	py	Ryd(2p)	0.00078	2.18228
373	H	35	pz	Ryd(2p)	0.0002	1.7137
**374**	**H**	**36**	**S**	**Val(1S)**	**0.76289**	**0.08759**
375	H	36	S	Ryd(2S)	0.00271	0.52011
376	H	36	px	Ryd(2p)	0.0007	2.16992
377	H	36	py	Ryd(2p)	0.00026	1.78057
378	H	36	pz	Ryd(2p)	0.0002	1.72964
379	C	37	S	Cor(1S)	1.99927	-10.057
380	C	37	S	Val(2S)	1.09424	-0.2501
381	C	37	S	Ryd(3S)	0.00079	1.24614
**382**	**C**	**37**	**px**	**Val(2p)**	**1.10004**	**-0.0738**
383	C	37	px	Ryd(3p)	0.00251	0.52892
384	C	37	py	Val(2p)	1.21475	-0.0827
385	C	37	py	Ryd(3p)	0.00351	0.64033
386	C	37	pz	Val(2p)	1.21551	-0.082
387	C	37	pz	Ryd(3p)	0.00359	0.64909
388	C	37	dxy	Ryd(3d)	0.00041	1.77471
389	C	37	dxz	Ryd(3d)	0.00048	1.82819
390	C	37	dyz	Ryd(3d)	0.00012	1.73777

179	C	15	dxy	Ryd(3d)	0.00079	1.91264
180	C	15	dxz	Ryd(3d)	0.00043	1.76677
181	C	15	dyz	Ryd(3d)	0.00063	1.73818
182	C	15	dx2y2	Ryd(3d)	0.00062	1.80905
183	C	15	dz2	Ryd(3d)	0.00062	1.95025
184	C	16	S	Cor(1S)	1.99909	-10.05
185	C	16	S	Val(2S)	1.04388	-0.226
186	C	16	S	Ryd(3S)	0.00169	1.4219
187	C	16	px	Val(2p)	1.15399	-0.0832
188	C	16	px	Ryd(3p)	0.00447	0.7179
189	C	16	py	Val(2p)	1.22419	-0.0839
190	C	16	py	Ryd(3p)	0.0061	0.79605
**191**	**C**	**16**	**pz**	**Val(2p)**	**1.07545**	**-0.0717**
192	C	16	pz	Ryd(3p)	0.0045	0.61412
193	C	16	dxy	Ryd(3d)	0.0005	1.76117
194	C	16	dxz	Ryd(3d)	0.00065	1.94599
195	C	16	dyz	Ryd(3d)	0.00057	1.75518
196	C	16	dx2y2	Ryd(3d)	0.00049	1.91253
197	C	16	dz2	Ryd(3d)	0.00061	1.82812
**198**	**H**	**17**	**S**	**Val(1S)**	**0.57354**	**0.08451**
199	H	17	S	Ryd(2S)	0.00371	0.42858
200	H	17	px	Ryd(2p)	0.00064	1.80212
201	H	17	py	Ryd(2p)	0.00056	1.86423
202	H	17	pz	Ryd(2p)	0.00125	2.01146
203	C	18	S	Cor(1S)	1.99903	-10.073
391	C	37	dx2y2	Ryd(3d)	0.00064	1.89143
392	C	37	dz2	Ryd(3d)	0.00056	1.91633
**393**	**H**	**38**	**S**	**Val(1S)**	**0.77004**	**0.06778**
394	H	38	S	Ryd(2S)	0.0015	0.39353
395	H	38	px	Ryd(2p)	0.00014	1.69314
396	H	38	py	Ryd(2p)	0.00081	2.18226
397	H	38	pz	Ryd(2p)	0.00024	1.72627
**398**	**H**	**39**	**S**	**Val(1S)**	**0.77639**	**0.06277**
399	H	39	S	Ryd(2S)	0.00165	0.39365
400	H	39	px	Ryd(2p)	0.00016	1.70962
401	H	39	py	Ryd(2p)	0.00024	1.72588
402	H	39	pz	Ryd(2p)	0.00078	2.15447
**403**	**H**	**40**	**S**	**Val(1S)**	**0.77734**	**0.0635**
404	H	40	S	Ryd(2S)	0.00154	0.39932
405	H	40	px	Ryd(2p)	0.00049	1.9544
406	H	40	py	Ryd(2p)	0.00031	1.79542
407	H	40	pz	Ryd(2p)	0.00038	1.84789
**408**	**H**	**41**	**S**	**Val(1S)**	**0.74611**	**0.08521**
409	H	41	S	Ryd(2S)	0.00339	0.41097
410	H	41	px	Ryd(2p)	0.00019	1.80396
411	H	41	py	Ryd(2p)	0.00078	2.19074
412	H	41	pz	Ryd(2p)	0.00015	1.77135
**413**	**H**	**42**	**S**	**Val(1S)**	**0.74756**	**0.05674**

204	C	18	S	Val(2S)	0.90364	-0.2034		414	H	42	S	Ryd(2S)	0.00246	0.40233
205	C	18	S	Ryd(3S)	0.00032	1.54628		415	H	42	px	Ryd(2p)	0.00062	2.03079
206	C	18	px	Val(2p)	1.06045	-0.0956		416	H	42	py	Ryd(2p)	0.00025	1.84512
207	C	18	px	Ryd(3p)	0.00462	0.89495		417	H	42	pz	Ryd(2p)	0.00031	1.84162
**208**	**C**	**18**	**py**	**Val(2p)**	**1.02318**	**-0.0887**								
209	C	18	py	Ryd(3p)	0.00254	0.74748		**418**	**H**	**43**	**S**	**Val(1S)**	**0.743**	**0.08863**
210	C	18	pz	Val(2p)	1.04834	-0.0909		419	H	43	S	Ryd(2S)	0.00413	0.41635
211	C	18	pz	Ryd(3p)	0.00243	0.74599		420	H	43	px	Ryd(2p)	0.00019	1.8431
212	C	18	dxy	Ryd(3d)	0.00088	1.78777		421	H	43	py	Ryd(2p)	0.00012	1.85868
213	C	18	dxz	Ryd(3d)	0.00098	1.7998		422	H	43	pz	Ryd(2p)	0.00085	2.23222
214	C	18	dyz	Ryd(3d)	0.0008	1.73237								
215	C	18	dx2y2	Ryd(3d)	0.00125	1.89883								
216	C	18	dz2	Ryd(3d)	0.00128	1.91178								
**4b**														
1	C	1	S	Cor(1S)	1.99859	-10.123		**217**	**H**	**19**	**S**	**Val(1S)**	**0.75786**	**0.04795**
2	C	1	S	Val(2S)	0.82303	-0.115		218	H	19	S	Ryd(2S)	0.00258	0.41152
3	C	1	S	Ryd(3S)	0.00192	1.034		219	H	19	px	Ryd(2p)	0.0002	1.79765
4	C	1	px	Val(2p)	1.05657	-0.0744		220	H	19	py	Ryd(2p)	0.00015	1.77123
5	C	1	px	Ryd(3p)	0.00334	0.79287		221	H	19	pz	Ryd(2p)	0.0008	2.12992
6	C	1	py	Val(2p)	1.05628	-0.1071								
7	C	1	py	Ryd(3p)	0.00182	0.64654		**222**	**H**	**20**	**S**	**Val(1S)**	**0.75043**	**0.06231**
**8**	**C**	**1**	**pz**	**Val(2p)**	**0.76881**	**-0.026**		223	H	20	S	Ryd(2S)	0.00203	0.41336
9	C	1	pz	Ryd(3p)	0.00917	0.77386		224	H	20	px	Ryd(2p)	0.00058	2.01071
10	C	1	dxy	Ryd(3d)	0.00071	1.86579		225	H	20	py	Ryd(2p)	0.00028	1.81984
11	C	1	dxz	Ryd(3d)	0.00148	1.91078		226	H	20	pz	Ryd(2p)	0.00038	1.91171
12	C	1	dyz	Ryd(3d)	0.00231	1.63902								
13	C	1	dx2y2	Ryd(3d)	0.0008	1.64699		227	O	21	S	Cor(1S)	1.99972	-18.988
14	C	1	dz2	Ryd(3d)	0.002	2.02422		228	O	21	S	Val(2S)	1.59314	-0.887

15	C	2	S	Cor(1S)	1.9985	-10.118
16	C	2	S	Val(2S)	0.812	-0.1
17	C	2	S	Ryd(3S)	0.00182	1.12004
18	C	2	px	Val(2p)	0.96442	-0.0578
19	C	2	px	Ryd(3p)	0.00675	0.81767
20	C	2	py	Val(2p)	0.96208	-0.0968
21	C	2	py	Ryd(3p)	0.00314	0.65452
**22**	**C**	**2**	**pz**	**Val(2p)**	**0.90808**	**-0.0396**
23	C	2	pz	Ryd(3p)	0.00618	0.77204
24	C	2	dxy	Ryd(3d)	0.00104	1.84426
25	C	2	dxz	Ryd(3d)	0.00186	1.99113
26	C	2	dyz	Ryd(3d)	0.00203	1.67599
27	C	2	dx2y2	Ryd(3d)	0.00151	1.65403
28	C	2	dz2	Ryd(3d)	0.00158	2.01073
29	C	3	S	Cor(1S)	1.99882	-10.053
30	C	3	S	Val(2S)	0.87639	-0.1167
31	C	3	S	Ryd(3S)	0.00191	1.07881
32	C	3	px	Val(2p)	1.05916	-0.0629
33	C	3	px	Ryd(3p)	0.00667	0.97912
34	C	3	py	Val(2p)	1.06355	-0.0876
35	C	3	py	Ryd(3p)	0.00353	0.76345
**36**	**C**	**3**	**pz**	**Val(2p)**	**1.08567**	**-0.0503**
37	C	3	pz	Ryd(3p)	0.00449	0.97815
38	C	3	dxy	Ryd(3d)	0.0006	1.86526
39	C	3	dxz	Ryd(3d)	0.00072	1.98183
40	C	3	dyz	Ryd(3d)	0.00068	1.73409

229	O	21	S	Ryd(3S)	0.00038	2.10909
230	O	21	px	Val(2p)	1.61277	-0.3346
231	O	21	px	Ryd(3p)	0.00161	1.37724
**232**	**O**	**21**	**py**	**Val(2p)**	**1.81701**	**-0.3305**
233	O	21	py	Ryd(3p)	0.00224	1.14439
234	O	21	pz	Val(2p)	1.54518	-0.3378
235	O	21	pz	Ryd(3p)	0.00166	1.19948
236	O	21	dxy	Ryd(3d)	0.00064	2.82525
237	O	21	dxz	Ryd(3d)	0.00073	2.91638
238	O	21	dyz	Ryd(3d)	0.00068	2.77271
239	O	21	dx2y2	Ryd(3d)	0.00062	2.77858
240	O	21	dz2	Ryd(3d)	0.00122	3.08349
241	O	22	S	Cor(1S)	1.99977	-18.972
242	O	22	S	Val(2S)	1.658	-0.9048
243	O	22	S	Ryd(3S)	0.00015	2.22339
244	O	22	px	Val(2p)	1.74649	-0.3302
245	O	22	px	Ryd(3p)	0.00142	1.24909
246	O	22	py	Val(2p)	1.82253	-0.3198
247	O	22	py	Ryd(3p)	0.00184	1.08543
**248**	**O**	**22**	**pz**	**Val(2p)**	**1.46103**	**-0.3061**
249	O	22	pz	Ryd(3p)	0.00227	1.25187
250	O	22	dxy	Ryd(3d)	0.00111	2.85341
251	O	22	dxz	Ryd(3d)	0.00134	3.24365
252	O	22	dyz	Ryd(3d)	0.00036	2.86231
253	O	22	dx2y2	Ryd(3d)	0.00121	2.82367
254	O	22	dz2	Ryd(3d)	0.00042	3.05468

41	C	3	dx2y2	Ryd(3d)	0.00071	1.66616
42	C	3	dz2	Ryd(3d)	0.00065	2.0778
43	C	4	S	Cor(1S)	1.99891	-10.045
44	C	4	S	Val(2S)	0.94618	-0.1326
45	C	4	S	Ryd(3S)	0.00113	1.06144
**46**	**C**	**4**	**px**	**Val(2p)**	**1.06253**	**-0.0444**
47	C	4	px	Ryd(3p)	0.00343	0.71293
48	C	4	py	Val(2p)	1.01973	-0.0783
49	C	4	py	Ryd(3p)	0.00178	0.59955
50	C	4	pz	Val(2p)	1.17115	-0.0486
51	C	4	pz	Ryd(3p)	0.00617	0.95402
52	C	4	dxy	Ryd(3d)	0.00049	1.88803
53	C	4	dxz	Ryd(3d)	0.00061	1.96833
54	C	4	dyz	Ryd(3d)	0.00051	1.64354
55	C	4	dx2y2	Ryd(3d)	0.00063	1.68706
56	C	4	dz2	Ryd(3d)	0.00052	2.09238
57	C	5	S	Cor(1S)	1.99899	-10.05
58	C	5	S	Val(2S)	0.95158	-0.1404
59	C	5	S	Ryd(3S)	0.00096	1.0184
60	C	5	px	Val(2p)	1.09192	-0.049
61	C	5	px	Ryd(3p)	0.00409	0.70923
62	C	5	py	Val(2p)	1.05962	-0.0841
63	C	5	py	Ryd(3p)	0.00233	0.64247
**64**	**C**	**5**	**pz**	**Val(2p)**	**1.11327**	**-0.0394**
65	C	5	pz	Ryd(3p)	0.00497	0.82003
66	C	5	dxy	Ryd(3d)	0.00047	1.83118

**255**	**H**	**23**	**S**	**Val(1S)**	**0.50103**	**0.09594**
256	H	23	S	Ryd(2S)	0.00294	0.4354
257	H	23	px	Ryd(2p)	0.00152	1.93448
258	H	23	py	Ryd(2p)	0.00147	1.57704
259	H	23	pz	Ryd(2p)	0.00212	2.17463
260	C	24	S	Cor(1S)	1.99935	-10.155
261	C	24	S	Val(2S)	1.10803	-0.2873
262	C	24	S	Ryd(3S)	0.00156	1.07215
**263**	**C**	**24**	**px**	**Val(2p)**	**0.8347**	**-0.0794**
264	C	24	px	Ryd(3p)	0.00256	0.47189
265	C	24	py	Val(2p)	1.13013	-0.0985
266	C	24	py	Ryd(3p)	0.001	0.5098
267	C	24	pz	Val(2p)	1.16588	-0.1079
268	C	24	pz	Ryd(3p)	0.00294	0.6839
269	C	24	dxy	Ryd(3d)	0.00148	1.70277
270	C	24	dxz	Ryd(3d)	0.00111	1.71599
271	C	24	dyz	Ryd(3d)	0.00053	1.64519
272	C	24	dx2y2	Ryd(3d)	0.00162	1.78056
273	C	24	dz2	Ryd(3d)	0.001	1.89294
**274**	**H**	**25**	**S**	**Val(1S)**	**0.80452**	**0.02987**
275	H	25	S	Ryd(2S)	0.00272	0.36229
276	H	25	px	Ryd(2p)	0.00052	1.94119
277	H	25	py	Ryd(2p)	0.00038	1.72877
278	H	25	pz	Ryd(2p)	0.00035	1.74099
**279**	**H**	**26**	**S**	**Val(1S)**	**0.7847**	**0.04584**

67	C	5	dxz	Ryd(3d)	0.00061	2.06817		280	H	26	S	Ryd(2S)	0.0008	0.31004
68	C	5	dyz	Ryd(3d)	0.00059	1.70444		281	H	26	px	Ryd(2p)	0.0001	1.58596
69	C	5	dx2y2	Ryd(3d)	0.00054	1.63868		282	H	26	py	Ryd(2p)	0.00023	1.62243
70	C	5	dz2	Ryd(3d)	0.00054	1.94865		283	H	26	pz	Ryd(2p)	0.00087	2.15256
71	C	6	S	Cor(1S)	1.99892	-10.057		**284**	**H**	**27**	**S**	**Val(1S)**	**0.80528**	**0.0294**
72	C	6	S	Val(2S)	0.95712	-0.1511		285	H	27	S	Ryd(2S)	0.0027	0.36275
73	C	6	S	Ryd(3S)	0.00104	1.01646		286	H	27	px	Ryd(2p)	0.00014	1.59583
74	C	6	px	Val(2p)	1.16139	-0.073		287	H	27	py	Ryd(2p)	0.00085	2.14139
75	C	6	px	Ryd(3p)	0.00426	0.87267		288	H	27	pz	Ryd(2p)	0.00025	1.67347
76	C	6	py	Val(2p)	1.08585	-0.0982								
77	C	6	py	Ryd(3p)	0.00216	0.64322		289	N	28	S	Cor(1S)	1.99907	-14.212
**78**	**C**	**6**	**pz**	**Val(2p)**	**1.08339**	**-0.049**		290	N	28	S	Val(2S)	1.23964	-0.5421
79	C	6	pz	Ryd(3p)	0.00312	0.70123		291	N	28	S	Ryd(3S)	0.00146	1.71813
80	C	6	dxy	Ryd(3d)	0.00039	1.85081		292	N	28	px	Val(2p)	1.48457	-0.2725
81	C	6	dxz	Ryd(3d)	0.00068	1.92817		293	N	28	px	Ryd(3p)	0.00289	1.11141
82	C	6	dyz	Ryd(3d)	0.0007	1.67099		294	N	28	py	Val(2p)	1.38	-0.2686
83	C	6	dx2y2	Ryd(3d)	0.00054	1.65141		295	N	28	py	Ryd(3p)	0.00247	1.1895
84	C	6	dz2	Ryd(3d)	0.00041	2.11559		**296**	**N**	**28**	**pz**	**Val(2p)**	**1.50458**	**-0.2716**
								297	N	28	pz	Ryd(3p)	0.00311	1.1344
**85**	**H**	**7**	**S**	**Val(1S)**	**0.76651**	**0.08042**		298	N	28	dxy	Ryd(3d)	0.0006	2.38881
86	H	7	S	Ryd(2S)	0.0017	0.32301		299	N	28	dxz	Ryd(3d)	0.0005	2.36202
87	H	7	px	Ryd(2p)	0.00035	1.94567		300	N	28	dyz	Ryd(3d)	0.0006	2.41125
88	H	7	py	Ryd(2p)	0.00035	1.71645		301	N	28	dx2y2	Ryd(3d)	0.00052	2.27001
89	H	7	pz	Ryd(2p)	0.00052	2.08355		302	N	28	dz2	Ryd(3d)	0.00036	2.145
**90**	**H**	**8**	**S**	**Val(1S)**	**0.76464**	**0.06564**		303	N	29	S	Cor(1S)	1.99918	-14.201
91	H	8	S	Ryd(2S)	0.00155	0.34741		304	N	29	S	Val(2S)	1.25725	-0.5401

92	H	8	px	Ryd(2p)	0.00073	2.07265
93	H	8	py	Ryd(2p)	0.00037	1.70892
94	H	8	pz	Ryd(2p)	0.00012	1.96773
95	C	9	S	Cor(1S)	1.999	-10.112
96	C	9	S	Val(2S)	0.92703	-0.1895
97	C	9	S	Ryd(3S)	0.00138	1.51295
98	C	9	px	Val(2p)	1.05126	-0.0849
99	C	9	px	Ryd(3p)	0.00284	0.60679
**100**	**C**	**9**	**py**	**Val(2p)**	**0.82715**	**-0.0628**
101	C	9	py	Ryd(3p)	0.00662	0.66632
102	C	9	pz	Val(2p)	1.20534	-0.1051
103	C	9	pz	Ryd(3p)	0.01129	0.9467
104	C	9	dxy	Ryd(3d)	0.00108	1.89492
105	C	9	dxz	Ryd(3d)	0.00056	1.89554
106	C	9	dyz	Ryd(3d)	0.0009	1.74443
107	C	9	dx2y2	Ryd(3d)	0.0018	1.88757
108	C	9	dz2	Ryd(3d)	0.00119	2.13535
109	C	10	S	Cor(1S)	1.99869	-10.046
110	C	10	S	Val(2S)	0.87791	-0.1166
111	C	10	S	Ryd(3S)	0.00242	1.18498
**112**	**C**	**10**	**px**	**Val(2p)**	**1.08442**	**-0.0762**
113	C	10	px	Ryd(3p)	0.00571	0.91928
114	C	10	py	Val(2p)	1.11711	-0.0787
115	C	10	py	Ryd(3p)	0.00461	0.96371
116	C	10	pz	Val(2p)	1.10918	-0.0833
117	C	10	pz	Ryd(3p)	0.00396	0.84746
305	N	29	S	Ryd(3S)	0.00199	1.63674
306	N	29	px	Val(2p)	1.52413	-0.2514
307	N	29	px	Ryd(3p)	0.00417	1.12761
**308**	**N**	**29**	**py**	**Val(2p)**	**1.34121**	**-0.2422**
309	N	29	py	Ryd(3p)	0.00245	1.2194
310	N	29	pz	Val(2p)	1.49273	-0.2606
311	N	29	pz	Ryd(3p)	0.00232	1.01521
312	N	29	dxy	Ryd(3d)	0.0006	2.36773
313	N	29	dxz	Ryd(3d)	0.00048	2.36367
314	N	29	dyz	Ryd(3d)	0.00052	2.34956
315	N	29	dx2y2	Ryd(3d)	0.0004	2.33122
316	N	29	dz2	Ryd(3d)	0.00072	2.19963
317	O	30	S	Cor(1S)	1.9998	-18.852
318	O	30	S	Val(2S)	1.69253	-0.8685
319	O	30	S	Ryd(3S)	0.00045	2.10863
**320**	**O**	**30**	**px**	**Val(2p)**	**1.64559**	**-0.246**
321	O	30	px	Ryd(3p)	0.00165	1.17735
322	O	30	py	Val(2p)	1.7238	-0.2495
323	O	30	py	Ryd(3p)	0.00188	1.17926
324	O	30	pz	Val(2p)	1.51609	-0.2655
325	O	30	pz	Ryd(3p)	0.00063	1.06921
326	O	30	dxy	Ryd(3d)	0.0007	2.84381
327	O	30	dxz	Ryd(3d)	0.00089	3.04624
328	O	30	dyz	Ryd(3d)	0.00094	3.0312
329	O	30	dx2y2	Ryd(3d)	0.00061	2.83003
330	O	30	dz2	Ryd(3d)	0.00152	2.93035

118	C	10	dxy	Ryd(3d)	0.00118	1.60508		**331**	**H**	**31**	**S**	**Val(1S)**	**0.76204**	**0.08425**
119	C	10	dxz	Ryd(3d)	0.00097	1.68512		332	H	31	S	Ryd(2S)	0.00221	0.35079
120	C	10	dyz	Ryd(3d)	0.00079	1.6636		333	H	31	px	Ryd(2p)	0.00023	1.92716
121	C	10	dx2y2	Ryd(3d)	0.00057	1.52531		334	H	31	py	Ryd(2p)	0.0002	1.6774
122	C	10	dz2	Ryd(3d)	0.00123	1.4181		335	H	31	pz	Ryd(2p)	0.00079	2.19739
123	C	11	S	Cor(1S)	1.99879	-10.115		336	C	32	S	Cor(1S)	1.99927	-10.055
124	C	11	S	Val(2S)	0.81829	-0.115		337	C	32	S	Val(2S)	1.09597	-0.2491
125	C	11	S	Ryd(3S)	0.00159	0.98739		338	C	32	S	Ryd(3S)	0.00111	1.22889
126	C	11	px	Val(2p)	1.00085	-0.0831		**339**	**C**	**32**	**px**	**Val(2p)**	**1.1655**	**-0.0736**
127	C	11	px	Ryd(3p)	0.00479	0.88882		340	C	32	px	Ryd(3p)	0.00288	0.6046
**128**	**C**	**11**	**py**	**Val(2p)**	**0.87711**	**-0.0417**		341	C	32	py	Val(2p)	1.20766	-0.08
129	C	11	py	Ryd(3p)	0.00594	0.76977		342	C	32	py	Ryd(3p)	0.00332	0.63661
130	C	11	pz	Val(2p)	0.99178	-0.0898		343	C	32	pz	Val(2p)	1.16518	-0.0778
131	C	11	pz	Ryd(3p)	0.004	0.73213		344	C	32	pz	Ryd(3p)	0.00294	0.59906
132	C	11	dxy	Ryd(3d)	0.00112	1.89004		345	C	32	dxy	Ryd(3d)	0.00029	1.75624
133	C	11	dxz	Ryd(3d)	0.00074	2.05112		346	C	32	dxz	Ryd(3d)	0.00039	1.76338
134	C	11	dyz	Ryd(3d)	0.00088	1.95108		347	C	32	dyz	Ryd(3d)	0.00021	1.71528
135	C	11	dx2y2	Ryd(3d)	0.00141	1.93035		348	C	32	dx2y2	Ryd(3d)	0.00062	1.97787
136	C	11	dz2	Ryd(3d)	0.00125	1.77891		349	C	32	dz2	Ryd(3d)	0.00064	1.95957
137	C	12	S	Cor(1S)	1.99906	-10.133		**350**	**H**	**33**	**S**	**Val(1S)**	**0.77896**	**0.06236**
138	C	12	S	Val(2S)	0.80889	-0.1137		351	H	33	S	Ryd(2S)	0.00151	0.39857
139	C	12	S	Ryd(3S)	0.0054	1.00135		352	H	33	px	Ryd(2p)	0.00018	1.71839
140	C	12	px	Val(2p)	0.87939	-0.0449		353	H	33	py	Ryd(2p)	0.00023	1.73273
141	C	12	px	Ryd(3p)	0.00761	0.77327		354	H	33	pz	Ryd(2p)	0.00074	2.14716
**142**	**C**	**12**	**py**	**Val(2p)**	**0.91004**	**-0.0282**								
143	C	12	py	Ryd(3p)	0.00822	0.69258		**355**	**H**	**34**	**S**	**Val(1S)**	**0.77229**	**0.06845**

144	C	12	pz	Val(2p)	0.78855	-0.0372
145	C	12	pz	Ryd(3p)	0.01389	0.66884
146	C	12	dxy	Ryd(3d)	0.00175	1.98516
147	C	12	dxz	Ryd(3d)	0.00196	1.90781
148	C	12	dyz	Ryd(3d)	0.00168	1.9657
149	C	12	dx2y2	Ryd(3d)	0.00203	1.84397
150	C	12	dz2	Ryd(3d)	0.0031	1.73911
151	C	13	S	Cor(1S)	1.99921	-10.183
152	C	13	S	Val(2S)	0.87192	-0.2103
153	C	13	S	Ryd(3S)	0.00207	0.73691
154	C	13	px	Val(2p)	0.86702	-0.0932
155	C	13	px	Ryd(3p)	0.00899	0.5612
156	C	13	py	Val(2p)	1.02641	-0.1501
157	C	13	py	Ryd(3p)	0.00867	0.80314
**158**	**C**	**13**	**pz**	**Val(2p)**	**0.87435**	**-0.0883**
159	C	13	pz	Ryd(3p)	0.01093	0.55928
160	C	13	dxy	Ryd(3d)	0.00314	1.63564
161	C	13	dxz	Ryd(3d)	0.00126	1.83548
162	C	13	dyz	Ryd(3d)	0.00285	1.69375
163	C	13	dx2y2	Ryd(3d)	0.00231	1.6855
164	C	13	dz2	Ryd(3d)	0.00268	1.58792
**165**	**H**	**14**	**S**	**Val(1S)**	**0.56854**	**0.09597**
166	H	14	S	Ryd(2S)	0.00391	0.42783
167	H	14	px	Ryd(2p)	0.00112	1.96158
168	H	14	py	Ryd(2p)	0.0007	1.98502
169	H	14	pz	Ryd(2p)	0.00064	1.80198

356	H	34	S	Ryd(2S)	0.00141	0.3973
357	H	34	px	Ryd(2p)	0.00017	1.71813
358	H	34	py	Ryd(2p)	0.0008	2.19398
359	H	34	pz	Ryd(2p)	0.00018	1.69536
**360**	**H**	**35**	**S**	**Val(1S)**	**0.76309**	**0.08431**
361	H	35	S	Ryd(2S)	0.00278	0.51886
362	H	35	px	Ryd(2p)	0.0007	2.16583
363	H	35	py	Ryd(2p)	0.00027	1.77845
364	H	35	pz	Ryd(2p)	0.0002	1.72733
365	C	36	S	Cor(1S)	1.99927	-10.06
366	C	36	S	Val(2S)	1.09427	-0.2532
367	C	36	S	Ryd(3S)	0.00078	1.24372
**368**	**C**	**36**	**px**	**Val(2p)**	**1.09973**	**-0.0767**
369	C	36	px	Ryd(3p)	0.00252	0.52694
370	C	36	py	Val(2p)	1.21705	-0.086
371	C	36	py	Ryd(3p)	0.00352	0.64012
372	C	36	pz	Val(2p)	1.21367	-0.0848
373	C	36	pz	Ryd(3p)	0.00358	0.6432
374	C	36	dxy	Ryd(3d)	0.00035	1.74711
375	C	36	dxz	Ryd(3d)	0.00054	1.85159
376	C	36	dyz	Ryd(3d)	0.00015	1.74505
377	C	36	dx2y2	Ryd(3d)	0.00066	1.90188
378	C	36	dz2	Ryd(3d)	0.0005	1.8877
**379**	**H**	**37**	**S**	**Val(1S)**	**0.76953**	**0.06518**
380	H	37	S	Ryd(2S)	0.00149	0.39108

170	C	15	S	Cor(1S)	1.99908	-10.076
171	C	15	S	Val(2S)	1.01857	-0.2387
172	C	15	S	Ryd(3S)	0.00165	1.46218
**173**	**C**	**15**	**px**	**Val(2p)**	**1.13234**	**-0.0999**
174	C	15	px	Ryd(3p)	0.00424	0.6838
175	C	15	py	Val(2p)	1.09538	-0.1004
176	C	15	py	Ryd(3p)	0.0042	0.5837
177	C	15	pz	Val(2p)	1.20387	-0.1051
178	C	15	pz	Ryd(3p)	0.00499	0.77707
179	C	15	dxy	Ryd(3d)	0.0007	1.87788
180	C	15	dxz	Ryd(3d)	0.00051	1.79216
181	C	15	dyz	Ryd(3d)	0.00071	1.76966
182	C	15	dx2y2	Ryd(3d)	0.00064	1.8249
183	C	15	dz2	Ryd(3d)	0.00052	1.88913
184	C	16	S	Cor(1S)	1.99909	-10.055
185	C	16	S	Val(2S)	1.04408	-0.2309
186	C	16	S	Ryd(3S)	0.00169	1.41763
187	C	16	px	Val(2p)	1.15354	-0.0879
188	C	16	px	Ryd(3p)	0.00448	0.71702
189	C	16	py	Val(2p)	1.22142	-0.0879
190	C	16	py	Ryd(3p)	0.00638	0.77857
**191**	**C**	**16**	**pz**	**Val(2p)**	**1.07951**	**-0.0773**
192	C	16	pz	Ryd(3p)	0.00417	0.61931
193	C	16	dxy	Ryd(3d)	0.00044	1.73423
194	C	16	dxz	Ryd(3d)	0.00072	1.96643
195	C	16	dyz	Ryd(3d)	0.00054	1.72676

381	H	37	px	Ryd(2p)	0.00014	1.68981
382	H	37	py	Ryd(2p)	0.00082	2.18873
383	H	37	pz	Ryd(2p)	0.00023	1.71527
**384**	**H**	**38**	**S**	**Val(1S)**	**0.77543**	**0.06062**
385	H	38	S	Ryd(2S)	0.00162	0.39121
386	H	38	px	Ryd(2p)	0.00017	1.70971
387	H	38	py	Ryd(2p)	0.00026	1.74616
388	H	38	pz	Ryd(2p)	0.00075	2.12664
**389**	**H**	**39**	**S**	**Val(1S)**	**0.77686**	**0.06094**
390	H	39	S	Ryd(2S)	0.00152	0.39679
391	H	39	px	Ryd(2p)	0.00049	1.95214
392	H	39	py	Ryd(2p)	0.00028	1.76833
393	H	39	pz	Ryd(2p)	0.00041	1.86899
**394**	**H**	**40**	**S**	**Val(1S)**	**0.74485**	**0.08132**
395	H	40	S	Ryd(2S)	0.0033	0.40695
396	H	40	px	Ryd(2p)	0.00019	1.80006
397	H	40	py	Ryd(2p)	0.00081	2.20258
398	H	40	pz	Ryd(2p)	0.00012	1.75101
**399**	**H**	**41**	**S**	**Val(1S)**	**0.74616**	**0.05358**
400	H	41	S	Ryd(2S)	0.00242	0.39925
401	H	41	px	Ryd(2p)	0.00062	2.02758
402	H	41	py	Ryd(2p)	0.00022	1.82454
403	H	41	pz	Ryd(2p)	0.00034	1.85336

196	C	16	dx2y2	Ryd(3d)	0.00055	1.93655
197	C	16	dz2	Ryd(3d)	0.00058	1.81577
**198**	**H**	**17**	**S**	**Val(1S)**	**0.56281**	**0.08295**
199	H	17	S	Ryd(2S)	0.00444	0.42389
200	H	17	px	Ryd(2p)	0.00066	1.81072
201	H	17	py	Ryd(2p)	0.00067	1.9201
202	H	17	pz	Ryd(2p)	0.00113	1.94328
203	C	18	S	Cor(1S)	1.99903	-10.077
204	C	18	S	Val(2S)	0.90394	-0.2076
205	C	18	S	Ryd(3S)	0.00032	1.54309
206	C	18	px	Val(2p)	1.06161	-0.0997
207	C	18	px	Ryd(3p)	0.00462	0.89097
**208**	**C**	**18**	**py**	**Val(2p)**	**1.02073**	**-0.0921**
209	C	18	py	Ryd(3p)	0.00251	0.73652
210	C	18	pz	Val(2p)	1.04918	-0.0954
211	C	18	pz	Ryd(3p)	0.00247	0.75087
212	C	18	dxy	Ryd(3d)	0.00082	1.75866
213	C	18	dxz	Ryd(3d)	0.00104	1.82403
214	C	18	dyz	Ryd(3d)	0.00082	1.73521
215	C	18	dx2y2	Ryd(3d)	0.00127	1.91121
216	C	18	dz2	Ryd(3d)	0.00124	1.88137

**404**	**H**	**42**	**S**	**Val(1S)**	**0.74018**	**0.08065**
405	H	42	S	Ryd(2S)	0.00407	0.40759
406	H	42	px	Ryd(2p)	0.00018	1.83624
407	H	42	py	Ryd(2p)	0.00012	1.85846
408	H	42	pz	Ryd(2p)	0.00085	2.21386
409	S	43	S	Cor(1S)	2	-87.596
410	S	43	S	Cor(2S)	1.99944	-8.8124
411	S	43	S	Val(3S)	1.76575	-0.7171
412	S	43	S	Ryd(4S)	0.00815	0.76895
413	S	43	px	Cor(2p)	1.99995	-5.8305
414	S	43	px	Val(3p)	1.70129	-0.1794
415	S	43	px	Ryd(4p)	0.00189	0.56415
416	S	43	py	Cor(2p)	1.99983	-5.835
**417**	**S**	**43**	**py**	**Val(3p)**	**1.08545**	**-0.1288**
418	S	43	py	Ryd(4p)	0.00528	0.51018
419	S	43	pz	Cor(2p)	1.99994	-5.831
420	S	43	pz	Val(3p)	1.65285	-0.1763
421	S	43	pz	Ryd(4p)	0.00189	0.55959
422	S	43	dxy	Ryd(3d)	0.00249	0.96106
423	S	43	dxz	Ryd(3d)	0.00052	0.73183
424	S	43	dyz	Ryd(3d)	0.00292	0.99554
425	S	43	dx2y2	Ryd(3d)	0.00389	0.97677
426	S	43	dz2	Ryd(3d)	0.00143	0.81077

Table S4. Non-bond interactions of molecules (4a and 4b) with proteins (PDB IDs: 1DB4 and 2PWB).

Distance (Å)	Category	Type	From	From Chemistry	To	To Chemistry
**Favourable Non-bond**						
**1DB4 vs 4a**						
2.27	Hydrogen Bond	Conventional Hydrogen Bond	A:SER113:HG	H-Donor	:UNK0:O	H-Acceptor
2.63	Hydrogen Bond	Conventional Hydrogen Bond	A:ASN114:HD 22	H-Donor	:UNK0:O	H-Acceptor
2.34	Hydrogen Bond	Conventional Hydrogen Bond	A:LYS115:HN	H-Donor	:UNK0:O	H-Acceptor
2.99	Hydrogen Bond	Conventional Hydrogen Bond	:UNK0:H	H-Donor	A:CYS28:O	H-Acceptor
3.52	Hydrophobic	Pi-Sigma	A:ASN114:CB	C-H	:UNK0	Pi-Orbitals
4.33	Hydrophobic	Alkyl	:UNK0:C	Alkyl	A:VAL30	Alkyl
3.90	Hydrophobic	Alkyl	:UNK0:C	Alkyl	A:VAL30	Alkyl
**1DB4 vs 4b**						
2.47	Hydrogen Bond	Conventional Hydrogen Bond	A:GLY29:HN	H-Donor	:UNK0:O	H-Acceptor
2.55	Hydrogen Bond	Conventional Hydrogen Bond	A:VAL30:HN	H-Donor	:UNK0:O	H-Acceptor
2.28	Hydrogen Bond	Conventional Hydrogen Bond	A:HIS47:HD1	H-Donor	:UNK0:O	H-Acceptor
3.70	Hydrogen Bond	Carbon Hydrogen Bond	:UNK0:C	H-Donor	A:HIS27:O	H-Acceptor
3.33	Hydrogen Bond	Carbon Hydrogen Bond	:UNK0:C	H-Donor	A:ASP48:O D2	H-Acceptor
5.09	Other	Pi-Sulfur	:UNK0:S	Sulfur	A:HIS6	Pi-Orbitals
5.23	Hydrophobic	Pi-Pi T-shaped	A:PHE5	Pi-Orbitals	:UNK0	Pi-Orbitals
4.92	Hydrophobic	Alkyl	:UNK0:C	Alkyl	A:LEU2	Alkyl
**2PWB vs 4a**						
2.07	Hydrogen Bond	Conventional Hydrogen Bond	A:TRP8:HN	H-Donor	:UNK0:O	H-Acceptor
2.41	Hydrogen Bond	Conventional Hydrogen Bond	A:ARG185:H H22	H-Donor	:UNK0:O	H-Acceptor
2.96	Hydrogen Bond	Conventional Hydrogen Bond	:UNK0:H	H-Donor	A:ASN5:O	H-Acceptor
2.61	Hydrogen Bond	Conventional Hydrogen Bond	:UNK0:H	H-Donor	A:ALA6:O	H-Acceptor

1.86	Hydrogen Bond	Conventional Hydrogen Bond	:UNK0:H	H-Donor	A:ALA6:O	H-Acceptor
3.18	Hydrogen Bond	Carbon Hydrogen Bond	A:TRP8:CD1	H-Donor	:UNK0:O	H-Acceptor
3.92	Hydrogen Bond;Electrostatic	Pi-Cation;Pi-Donor Hydrogen Bond	A:ARG185:NH1	Positive;H-Donor	:UNK0	Pi-Orbitals;Pi-Orbitals
4.69	Hydrophobic	Alkyl	:UNK0:C	Alkyl	A:LEU209	Alkyl
**2PWB vs 4b**						
2.23	Hydrogen Bond	Conventional Hydrogen Bond	A:VAL127:HN	H-Donor	:UNK0:O	H-Acceptor
2.05	Hydrogen Bond	Conventional Hydrogen Bond	A:ALA245:HN	H-Donor	:UNK0:O	H-Acceptor
2.95	Hydrogen Bond	Conventional Hydrogen Bond	:UNK0:H	H-Donor	A:GLY152:O	H-Acceptor
2.52	Hydrogen Bond	Conventional Hydrogen Bond	:UNK0:H	H-Donor	A:GLY152:O	H-Acceptor
2.73	Hydrogen Bond	Conventional Hydrogen Bond	:UNK0:H	H-Donor	:UNK0:O	H-Acceptor
3.41	Hydrogen Bond	Carbon Hydrogen Bond	A:GLY126:CA	H-Donor	:UNK0:O	H-Acceptor
3.41	Hydrogen Bond	Carbon Hydrogen Bond	A:GLY126:CA	H-Donor	:UNK0:O	H-Acceptor
3.26	Hydrogen Bond	Carbon Hydrogen Bond	:UNK0:C	H-Donor	A:LYS125:O	H-Acceptor
4.63	Hydrophobic	Pi-Alkyl	:UNK0	Pi-Orbitals	A:ALA245	Alkyl
**Unsatisfied Bond (within compounds 4a and4b)**						
**Name**	**Atom**	**Unsatisfied type**		**Name**	**Atom**	**Unsatisfied type**
**1DB4 vs 4a**						
:UNK0:H	H	Donor		:UNK0:H	H	Donor
:UNK0:O	O	Acceptor				
**1DB4 vs 4b**						
:UNK0:H	H	Donor		:UNK0:H	H	Donor
:UNK0:H	H	Donor				
**2PWB vs 4a**						
:UNK0:H	H	Donor		:UNK0:O	O	Acceptor
:UNK0:O	O	Acceptor				
**2PWB vs 4b**						

:UNK0:H	H	Donor		:UNK0:S	S	Donor

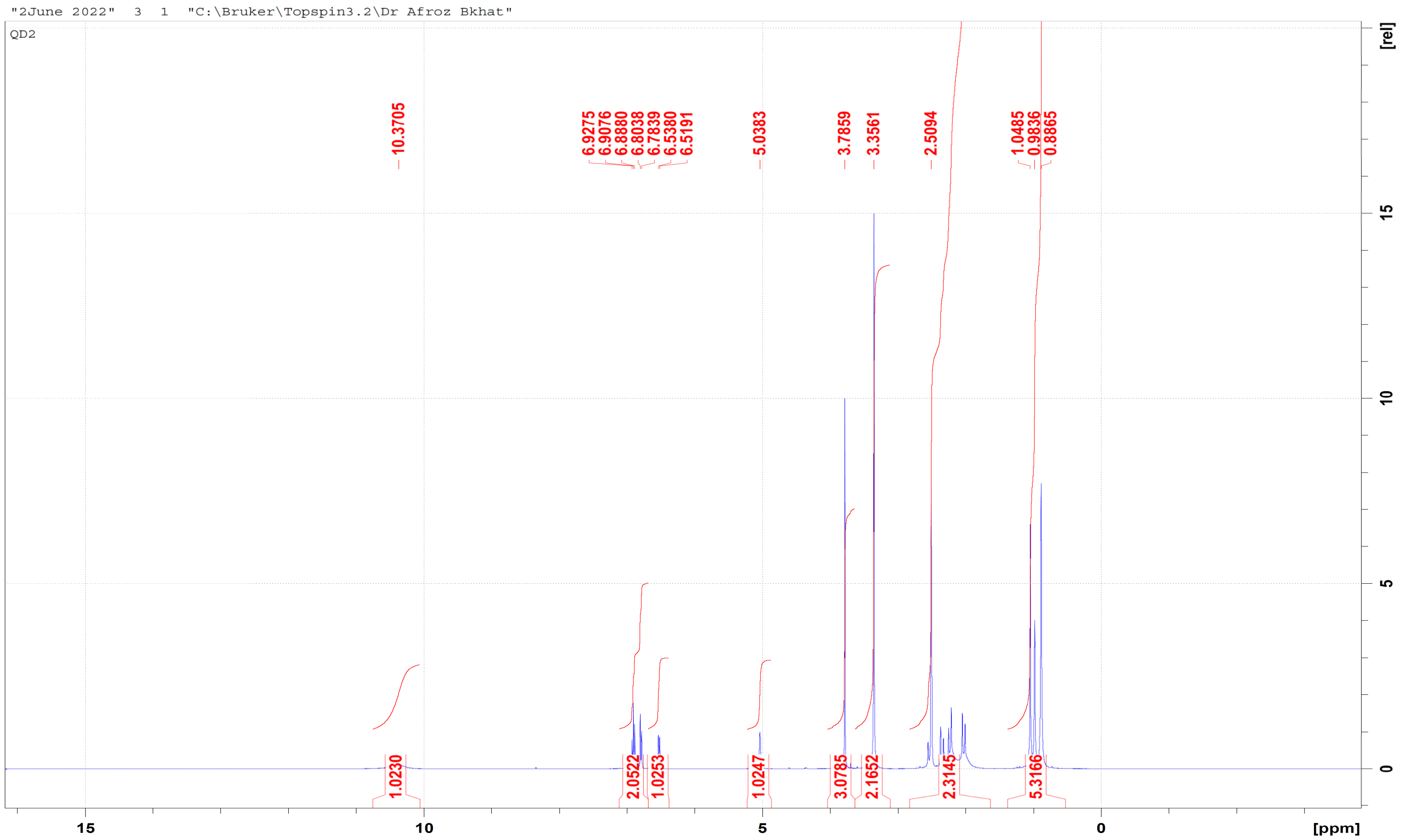

Figure S1. ^{1}H NMR of compound **4a**.

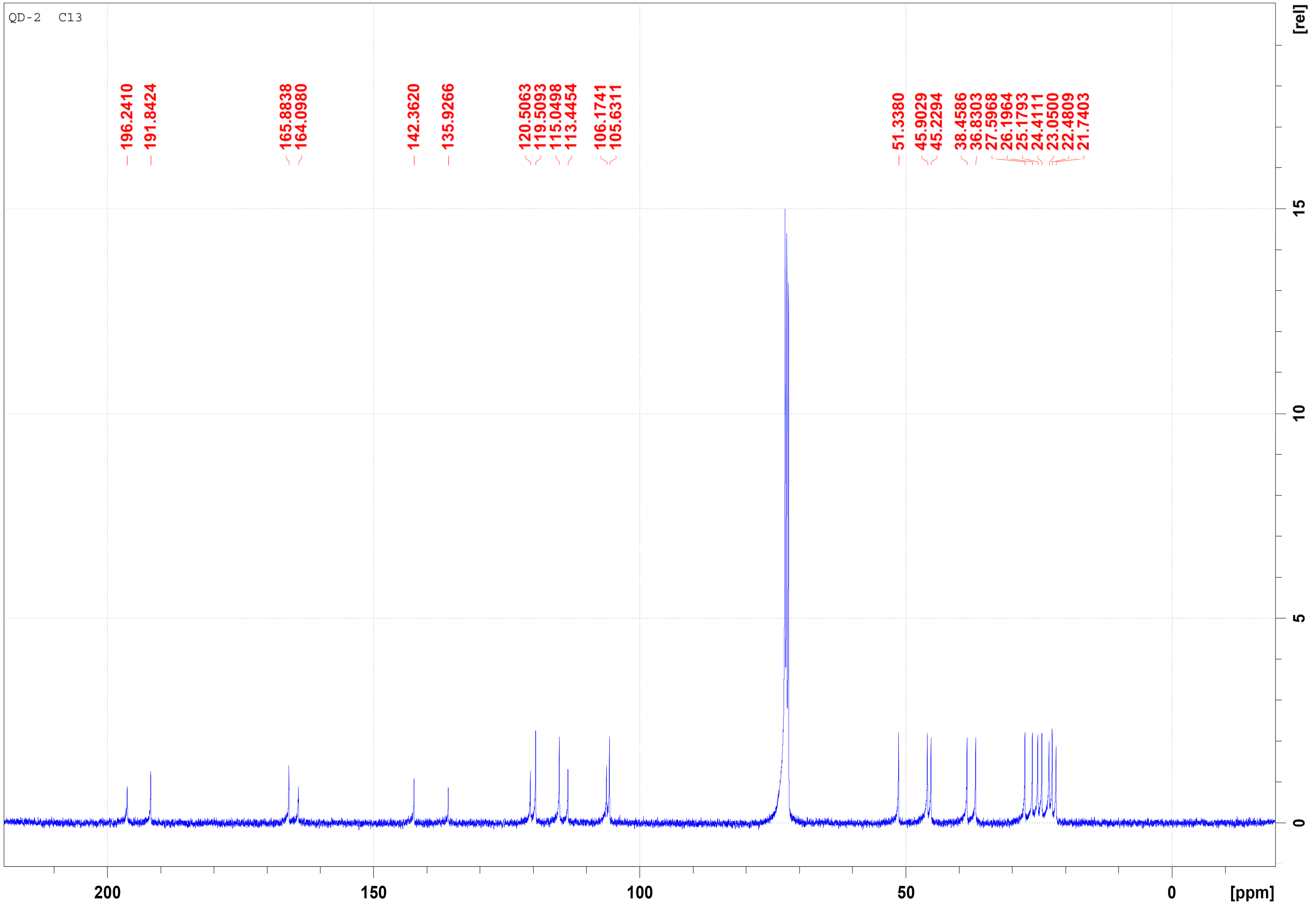

Figure S2. ^{13}C NMR of compound **4a**.

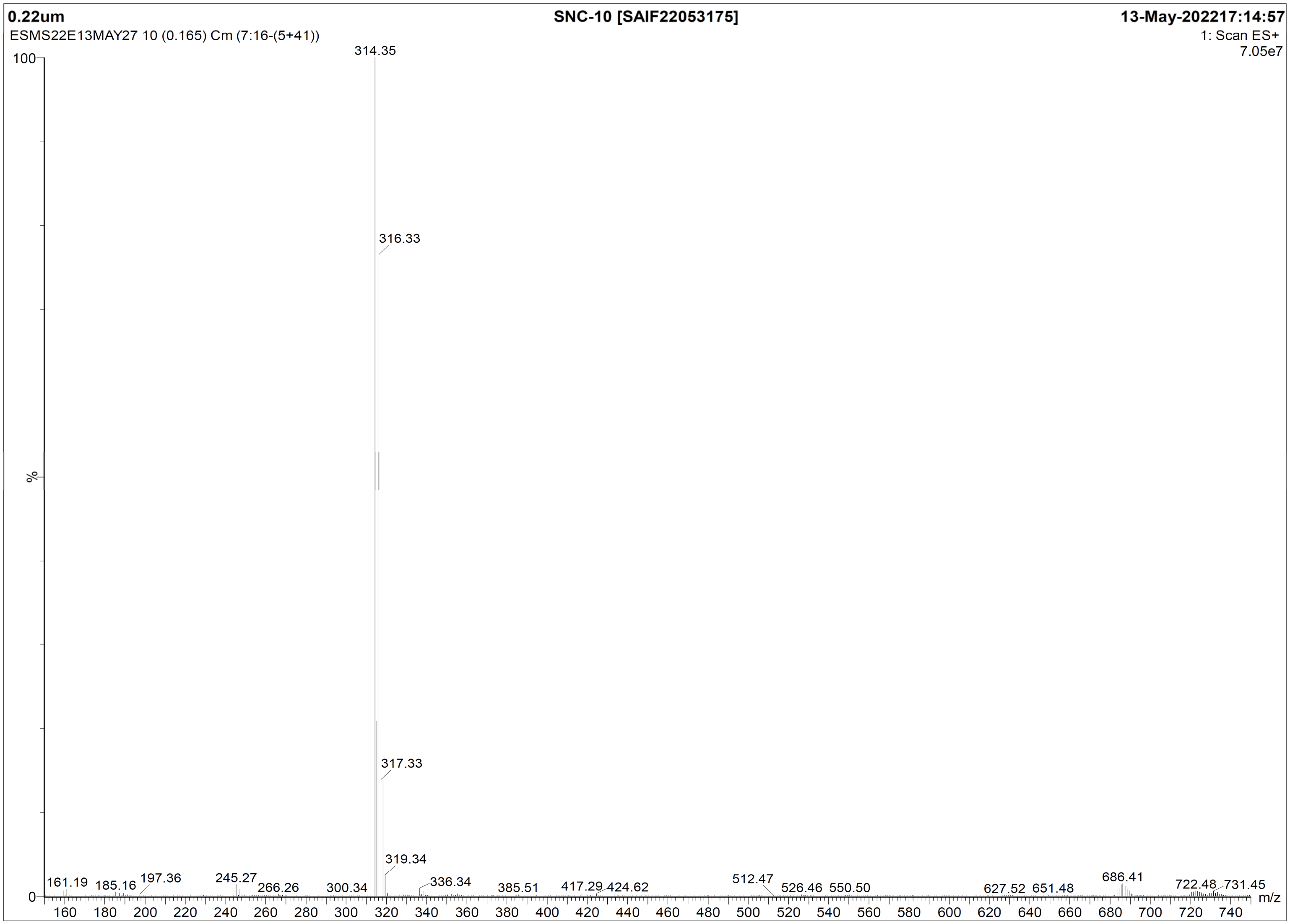

Figure S3. Mass spectra of compound **4a**.

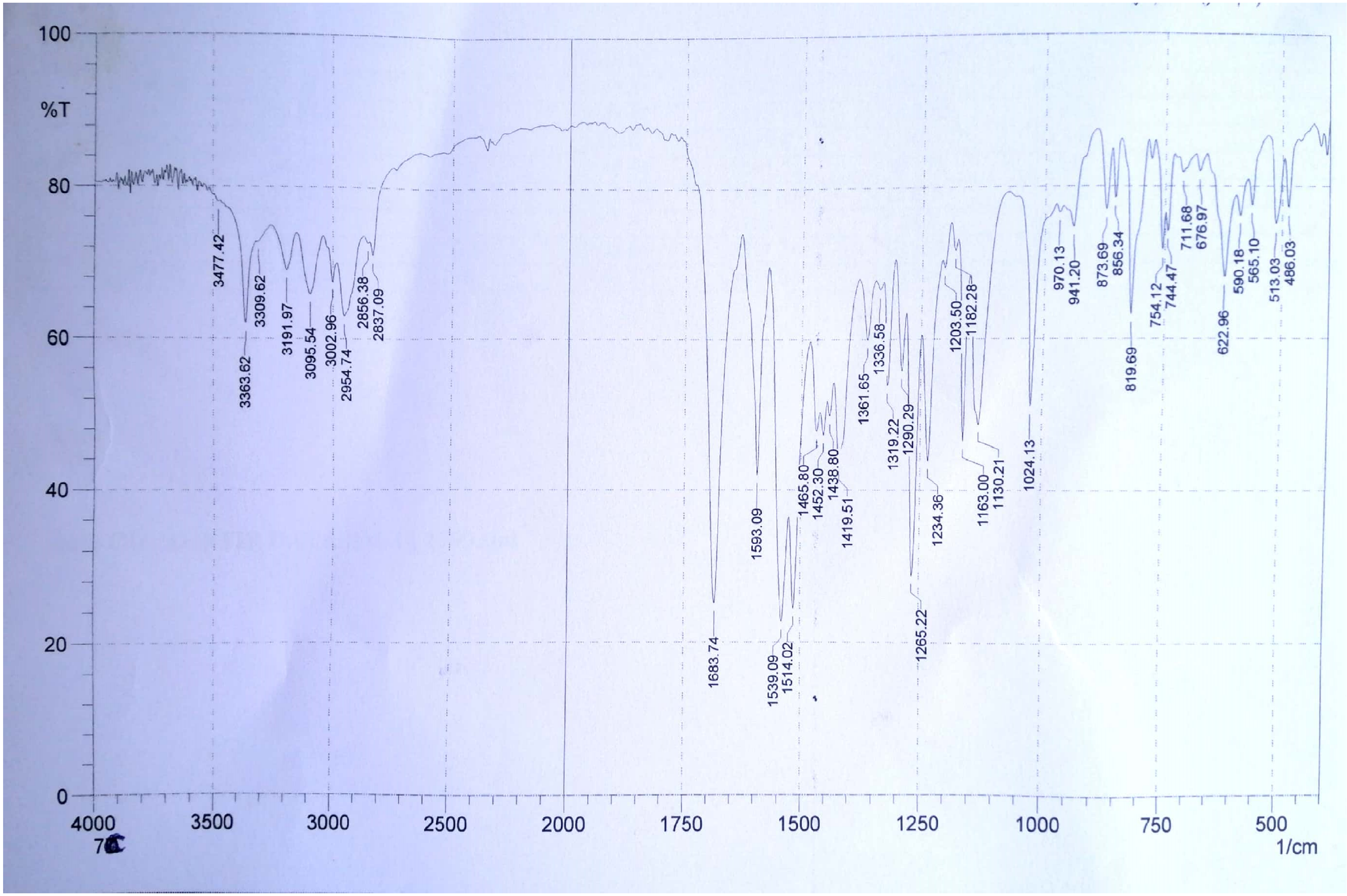

Figure S4. IR spectra of compound **4a**.

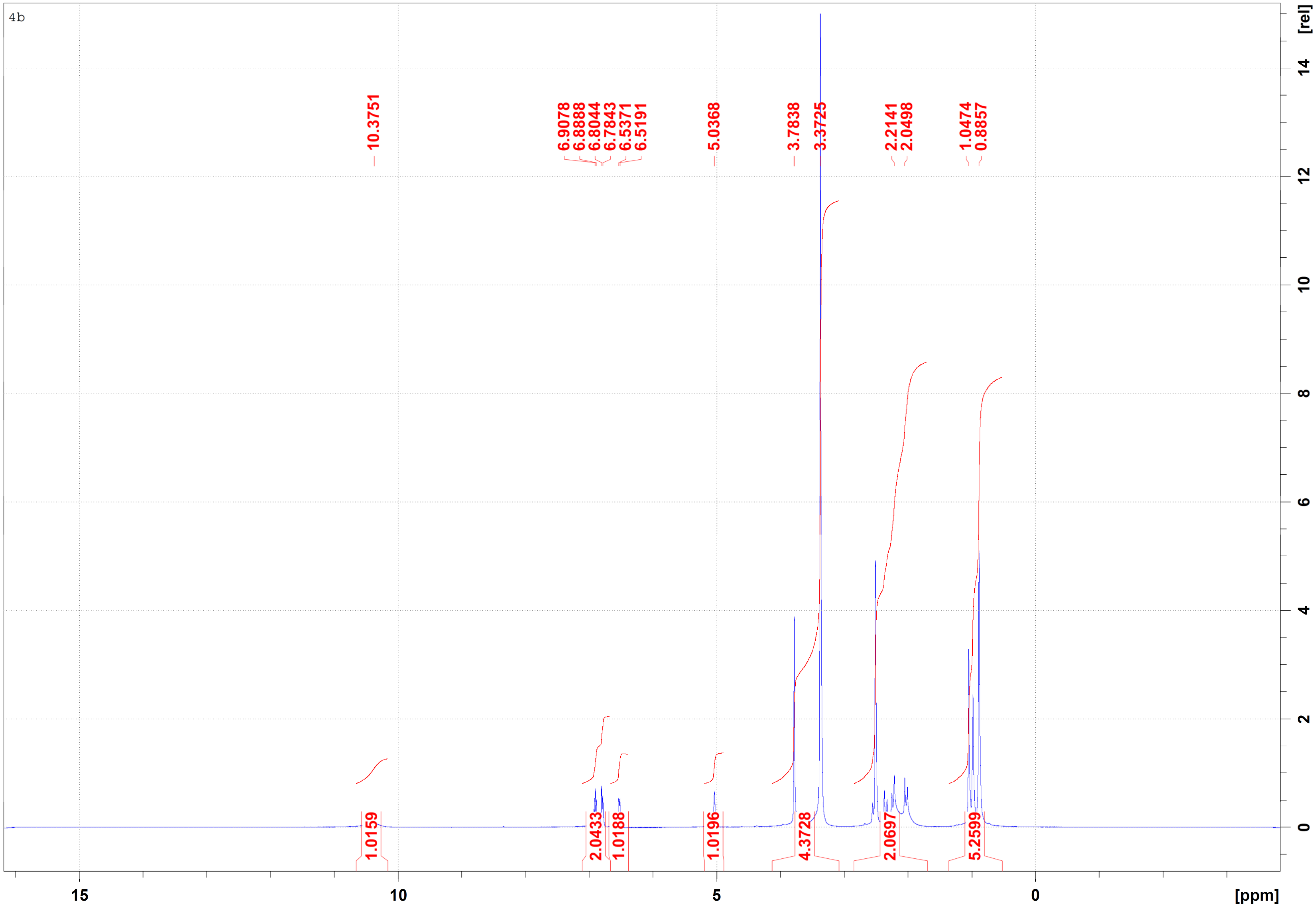

Figure S5. ^{1}H NMR spectra of compound **4b**.

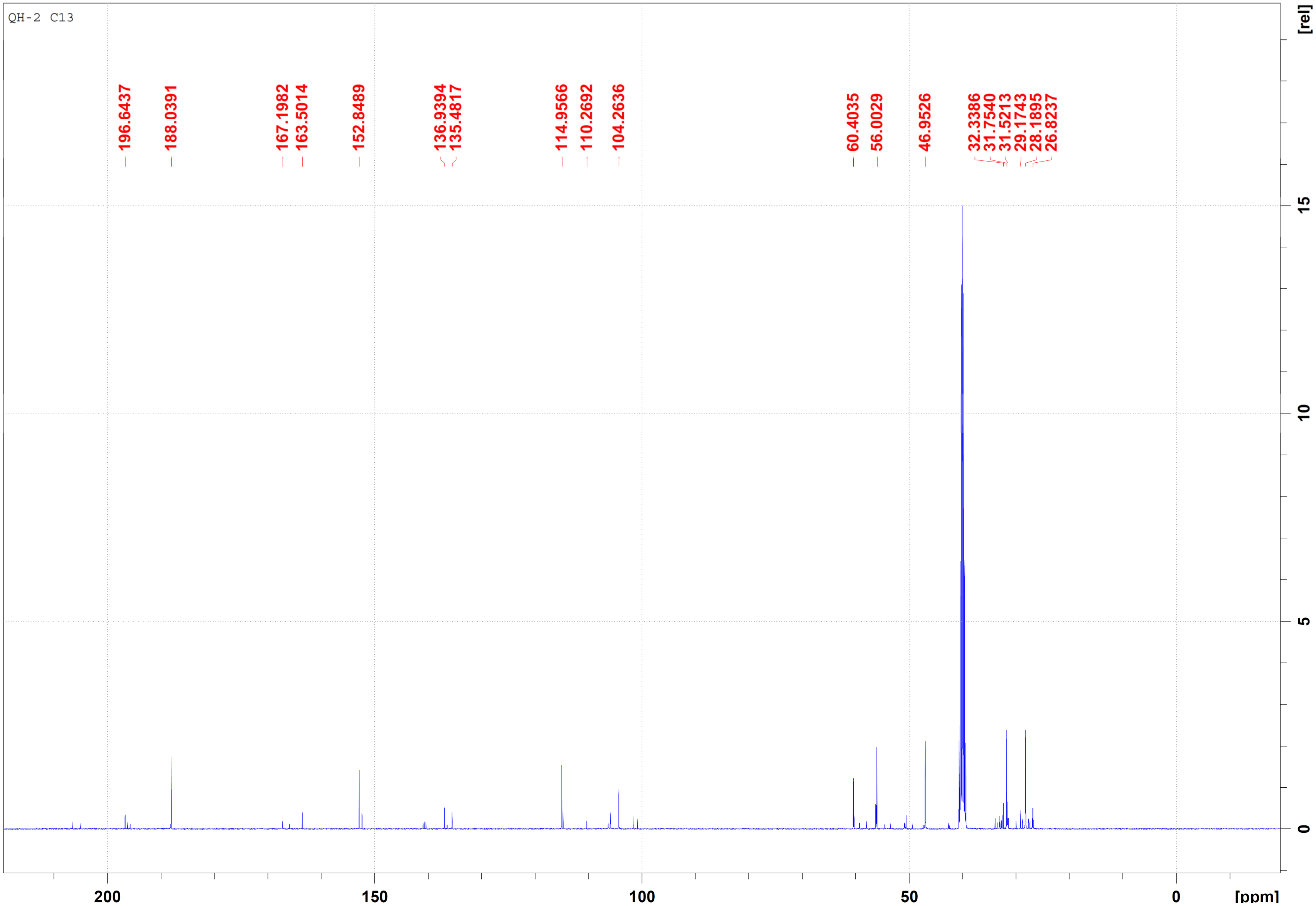

Figure S6. ^{13}C NMR spectra of compound **4b**.

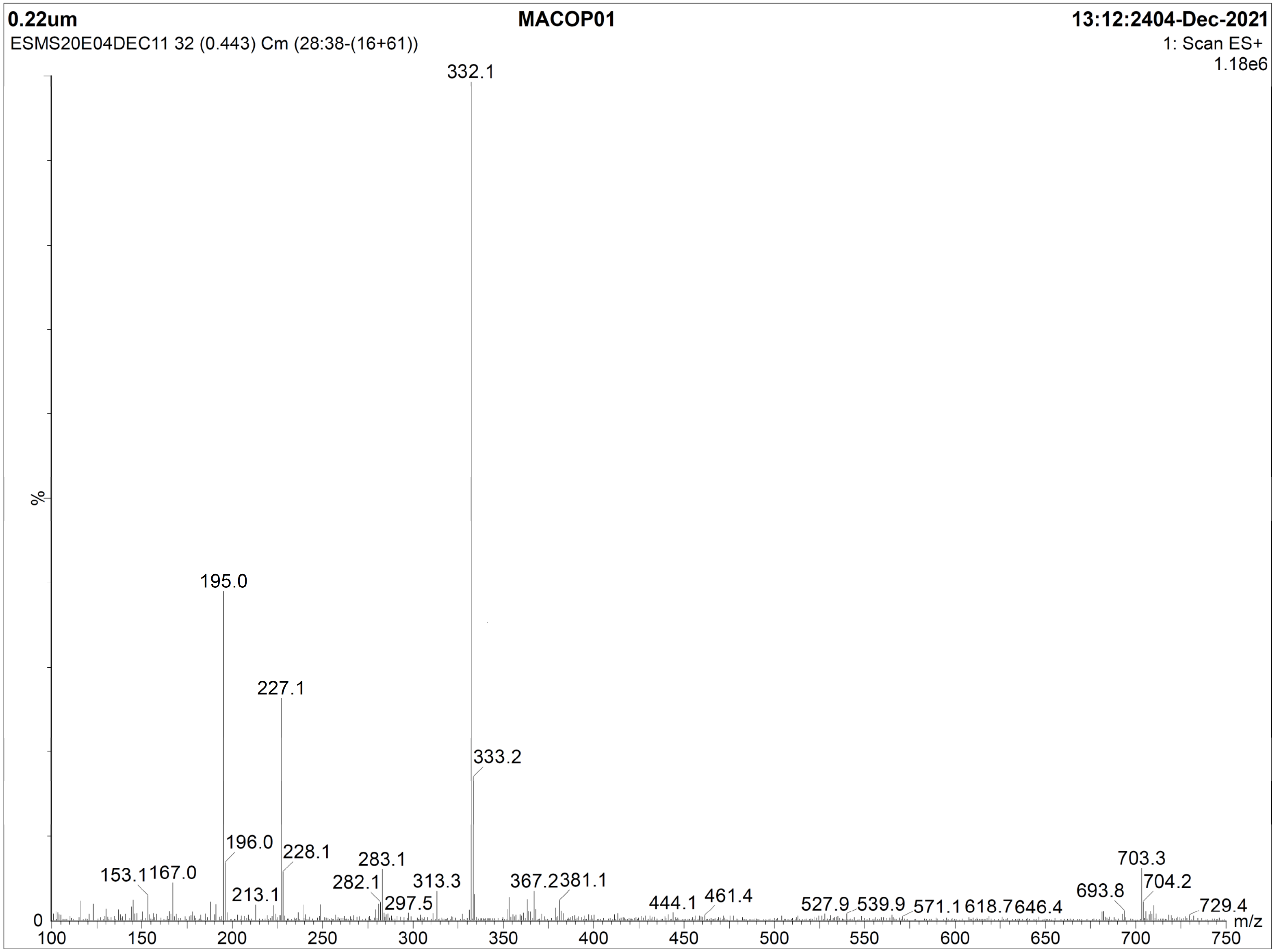

Figure S7. Mass spectra of compound **4b**.

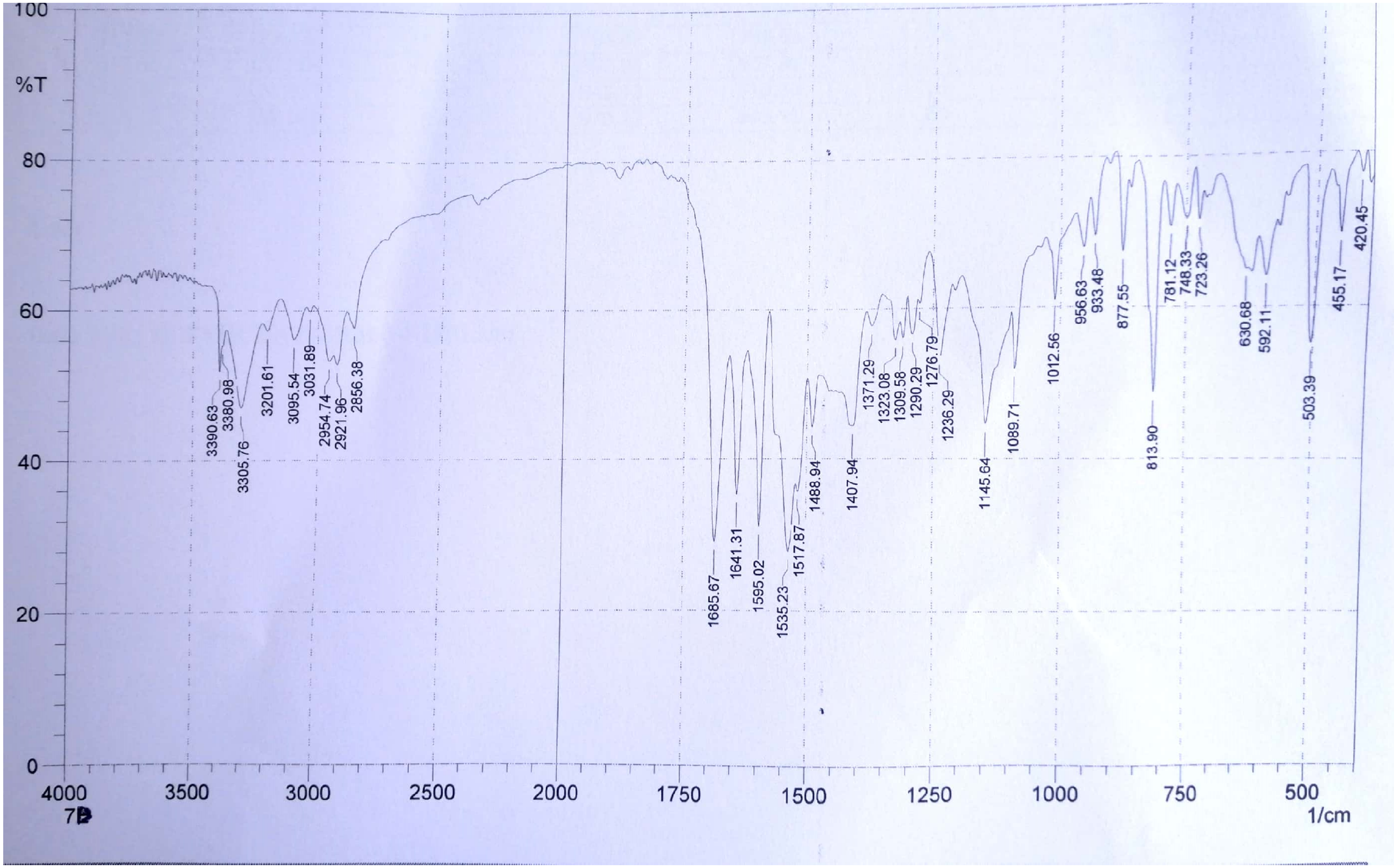

Figure S8. IR spectra of compound **4b**.
